# Gaussian Aquila optimizer based dual convolutional neural networks for identification and grading of osteoarthritis using knee joint images

**DOI:** 10.1038/s41598-024-57002-4

**Published:** 2024-03-27

**Authors:** B. Subha, Vijay Jeyakumar, S. N. Deepa

**Affiliations:** 1 Department of Biomedical Engineering, PSNA College of Engineering and Technology, Dindigul, India; 2 Department of Biomedical Engineering, Sri Sivasubramaniya Nadar College of Engineering, Chennai, India; 3 National Institute of Technology Calicut, NITC Campus Post, Kozhikode, Kerala India

**Keywords:** Knee-joint images, Osteoarthritis, Dual convolutional neural network, Gaussian mutation, Aquila optimizer, X-Ray images, Health care, Engineering

## Abstract

Degenerative musculoskeletal disease known as Osteoarthritis (OA) causes serious pain and abnormalities for humans and on detecting at an early stage, timely treatment shall be initiated to the patients at the earliest to overcome this pain. In this research study, X-ray images are captured from the humans and the proposed Gaussian Aquila Optimizer based Dual Convolutional Neural Networks is employed for detecting and classifying the osteoarthritis patients. The new Gaussian Aquila Optimizer (GAO) is devised to include Gaussian mutation at the exploitation stage of Aquila optimizer, which results in attaining the best global optimal value. Novel Dual Convolutional Neural Network (DCNN) is devised to balance the convolutional layers in each convolutional model and the weight and bias parameters of the new DCNN model are optimized using the developed GAO. The novelty of the proposed work lies in evolving a new optimizer, Gaussian Aquila Optimizer for parameter optimization of the devised DCNN model and the new DCNN model is structured to minimize the computational burden incurred in spite of it possessing dual layers but with minimal number of layers. The knee dataset comprises of total 2283 knee images, out of which 1267 are normal knee images and 1016 are the osteoarthritis images with an image of 512 × 512-pixel width and height respectively. The proposed novel GAO-DCNN system attains the classification results of 98.25% of sensitivity, 98.93% of specificity and 98.77% of classification accuracy for abnormal knee case–knee joint images. Experimental simulation results carried out confirms the superiority of the developed hybrid GAO-DCNN over the existing deep learning neural models form previous literature studies.

## Introduction

Osteoarthritis being a most prevalent degenerative musculoskeletal diseases is affecting almost 5% of the global population^1^. The human knees are the most common joints affected by osteoarthritis and is characterized by irreversible degeneration of the articular cartilage at the ends of the bones such as femoral, tibial, and patella cartilages. Knee osteoarthritis has been a progressive disease that affects the entire knee joint and is a condition driven by mechanical wear and tear as well as biochemical changes. Known risk factors for OA include aging, obesity and previous knee injuries^2–4^.

### Background of research study

**Figure 1:**
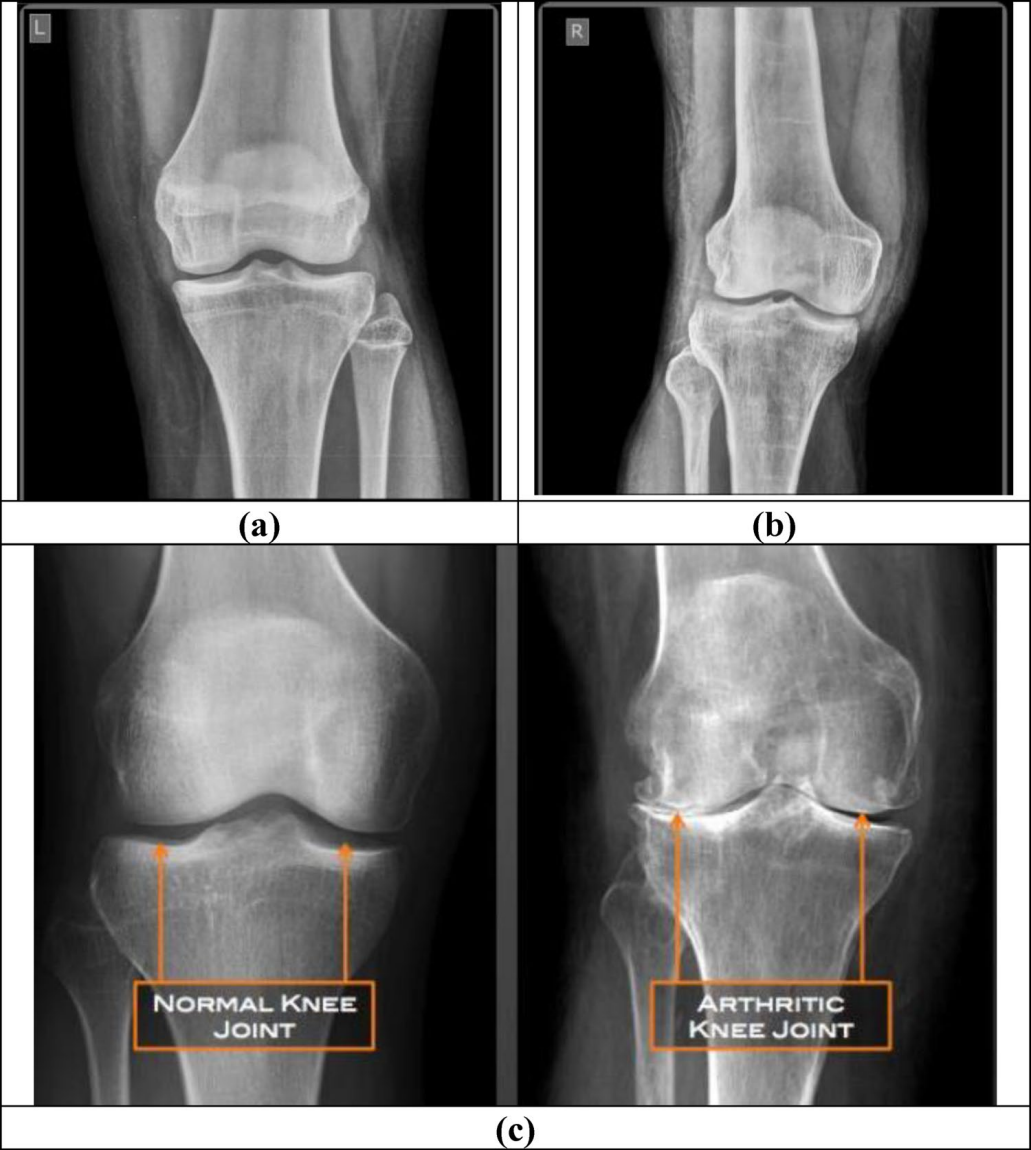
Knee Joint images (**a**) Normal image (**b**) Osteoarthritis image (**c**) Image with Markings.

The existence of osteoarthritis causes pain that limits function and reduces one’s quality of life. The joint damage of OA is irreversible, and definitive treatment requires total knee replacement (TKR), which is expensive and has a short life span especially for the obese individuals. Due to which, early detection of knee osteoarthritis is crucial for initiation of therapy including weight reduction and exercises that has been found to be effective in halting knee osteoarthritis progression and delaying the total knee replacement^3–6^. Figure 1 presents the normal knee image and the knee image affected with osteoarthritis. Radiographic grading scales for osteoarthritis rely primarily on Kellgren–Lawrence (KL) grading which examines the changes shown on X-ray plain radiography images. However, this approach causes delay in osteoarthritis diagnosis because the bone changes get appeared only in advanced conditions.

Besides X-ray, other imaging modalities such as magnetic resonance imaging can utilize several osteoarthritis of tissue biomarkers such as cartilage and meniscus degeneration and also deformation of the subchondral and trabecular bone to determine the onset of knee osteoarthritis^1^. There exists different types of osteoarthritis related segmentation or classification models for assessing the knee which are generally classified into classical methods and deep learning (DL) methods. In current clinical practices, evaluation of osteoarthritis severity is normally performed visually with radiography images, which is prone to interrater variability and time consumption for large datasets^7–9^.

Usually, osteoarthritis is observed with pain, stiffness and as well swelling over the peripheral joints. On leaving it without suitable treatment may result in irreversible joint disabilities, damages of joints and ligaments, quality of healthy life gets decreased. This may in worst case may lead to a higher mortality rate among the patients. All humans intend to perform more complex activities and as well basic movements and these activities has to be done individually by each and every person, resulting in a healthy life. On this getting disturbed, results in minimized physical functioning, depending others for activities to be done and gaining a long-term disorder for ease of movement. OA resulting in disability and chronic pain, has to be critically addressed and the methods for detecting and classifying its existence to be addressed appropriately to bring a healthy life of osteoarthritis patients and also to improve the socio- economic scenario across the globe. The World Health Organization (WHO) has several initiatives for classifying the functioning and disabilities noted with respect to osteoarthritis and various methods has been inculcated from 2001 onwards by WHO.

### Motivations and need for the study

There is always a requirement to develop and model suitable identification and classification models for the captured human knee joint images to detect the existence of osteoarthritis. It should be ascertained that the developed models are highly stable and reliable and attains the most possible level of accuracy so that the patients shall be treated in advance and their pain gets relieved. The need for osteoarthritis detection and classification at this present era is due to the following,

- Execution of daily living activities becomes difficult
- Longer time duration for curing
- Slower progression and reaction to the curing mechanism
- Prone to other health issues
- Activities and work limitations
- Severe OA results in malalignment of joints leading to surgeries
- Problem in the range of motion of the knee joints
- Difficulty in balancing and endurance ability
- Encounters problems in climbing stairs and lifting items

The above inconveniences experienced by the patients, result in the higher motivation of this research study for detecting and grading the osteoarthritis and based on the level of occurrence in the particular patient, the treatment gets initiated.

### Contributions

To determine the existence of osteoarthritis at the earliest stage and as well classifying its grade has been of high importance due to the higher populations of the human being prone to it. Henceforth is the development of this research study for identification and classification os osteoarthritis at an early stage. Considering this, the main contributions made in this paper includes,

- Modelling a new Gaussian mutation-based Aquila Optimizer (GAO) in the exploitation stage to attain the best global optimal values
- Design of Dual Convolutional Neural Network (DCNN) that performs the morphological feature extraction and classification of the normal and abnormal knee joint images in case of their existence
- Employing the new GAO into the DCNN model for tuning the weights of the fully connected layer of the dual convolutional neural learning model
- Applying the new hybrid GAO-DCNN for the captured human knee-joint images and evaluating the performance metrics
- To statistically validate the proposed hybrid GAO-DCNN model.

The developed dual convolutional neural network model with its optimal weight and bias coefficients tuned using the new Gaussian Aquila Optimizer enhanced the grading accuracy for the considered set of knee X-ray image datasets.

The remaining section of the paper is organized as follows: Section “Related works” provides the related works carried out in this area by previous researchers. The materials and methods developed for detection and classification of osteoarthritis is elucidated in section “Proposed methodology”. Section “Results and discussions” presents the experimental results attained and the discussions with respect to the results and their comparisons. Concluding remarks of the paper is given in section “Conclusion” of this paper.

## Related works

This section of the paper provides the early works carried out in detecting and classification of osteoarthritis using conventional and machine learning models. Kotti et al.^7^ used machine learning classification approach for grading the severity levels of the knee osteoarthritis using knee X-ray images. The authors computed discriminating feature set from the spatial domain knee image and these features were classified using Random Forest (RF) classification approach. This method was tested on several image dataset for validating the efficiency of the proposed knee grading system. Chen et al.^4^ used K-means clustering approach for detecting and segmenting the knee regions in source Knee images. The adjustable ordinal loss was computed for the detected knee point regions and from these segmented regions; the internal probabilistic features were computed for each internal layers of the designed Faster R-CNN deep learning architecture model. This method obtained 93.8% of GR for healthy knee image case, 95.3% of GR for Grade 1 OA, 96.1% of GR for Grade 2 OA and 95.9% of GR for Grade 3 OA.

Tiulpin and Saarakkala^2^ used deep Convolutional neural networks for identifying the level of severity grading using knee X-ray images. The data augmentation methods used in this work were random rotation and Gaussian noise addition on source knee images. The softmax layer was optimized using ReLU activation property which was designed by the Sigmoid function. This method used six numbers of neural networks layers at the output side of the CNN architecture. Liu et al.^3^ analyzed the severity of knee OA with respect to various grading using the modified Residual-CNN (RCNN) architecture model. The relevant Discriminant features were computed from each Convolution layer in the designed RCNN architecture and these internal layer features were used to classify the grades of the knee OA severity. Thomas et al.^1^ used deep learning CNN architecture model for detecting and grading the severity levels of the knee OA. The knee images from the open access dataset were data augmented to increase the sample sizes and the data augmented knee images were directly applied to the designed CNN architecture. The performance of the proposed knee OA grading method was analyzed using different number of internal layers with various neurons in fully connected layers.

Alexos et al.^10^ predicted the knee osteoarthritis abnormalities in X-ray images using SVM classification models. The authors performed extensive experimentation and validation on the large clinical X-ray image dataset to predict the knee osteoarthritis. Jamshidi et al.^11^ used various machine learning algorithms to locate the osteoarthritis abnormalities in X-ray images. The voting function was constructed between the applied machine learning algorithms and the suitable classifier was selected based on the voting values.The effect and applicability’s of various machine learning algorithms on the detection process of knee joint abnormalities were analyzed by researchers Teoh et al.^12^, Hafezi-Nejad et al.^13^, Tolpadi et al.^14^, Guan et al.^15^.

Yeoh et al.^16^ segmented the regions of knee Cartilages in the knee joint of the 3D-knee images for the detection of OA using CNN classification procedure. The CNN features were used in this work for the classification of knee images into either OA affected or not. Yifan Wang et al.^17^ used Kellgren–Lawrence (KL) grading system for the identification of OA using deep learning framework models in this work. The authors tested the developed OA detection process based on the segmented knee joint regions. Kokkotis et al.^18^ constructed fuzzy logic to extract the knee image features and these extracted feature set were used to identify the OA affected knee image from the normal healthy knee image. Various machine learning based and heuristic computational based solutions has also been provided in previous works for finding solutions to osteoarthritis problem^19–30^ and each of these methods had their own advantages and limitations.

Teoh et al.^31^ develop a multitask model using convolutional neural network (CNN) feature extractors and machine learning classifiers to detect nine important OA features and a new feature extraction method by replacing fully-connected layer with global average pooling (GAP) layer. Kijowski et al.^32^ presented a detailed review of current applications of DL in osteoarthritis (OA) imaging, including methods used for cartilage lesion detection, OA diagnosis, cartilage segmentation, and OA risk assessment. Ibraheem et al.^33^ developed a predictive model, called the Enhanced Sequencing Deep Learning (PESDL) Algorithm, to detect Patellofemoral osteoarthritis from the sEMG signal.

Hu et al.^34^ develop a potential deep*-*learning model for predicting OA progression based on MR images for the clinical setting. An X-ray-based model and traditional models that used clinical variables via multilayer perceptron were built. Combined models were also constructed, which integrated clinical variables with DeepKOA. Abd El-Ghany et al.^35^ proposed a fine-tuning KOA diagnosis model using the DenseNet169 deep learning (DL) technique to improve the efficiency of KOA diagnosis. The proposed model will determine the degree of KOA diseases by making multi-classification and binary classifications of the KOA severity^35^. Mahum et al.^36^ proposes a technique based on an efficient DenseNet that uses the knee image' features to identify the Knee Osteoarthritis (KOA) and determine its severity level according to the KL grading system such as Grade-I, Grade-II, Grade-III, and Grade-IV.

### Implications from the survey

Considering the literature review made in the detection and grading of knee osteoarthritis as carried out by previous researchers, it has been observed that there exist numerous conventional and computational intelligent based methods developed. Each of the present techniques employed for the detection and classification of this osteoarthritis has its own merits and demerits and certain limitations are presented as follows:

- Inherent biases in the clinical data (Liu et al.^3^, Kotti et al.^5^)
- Lack of external validation (Rutherford and Baker^22^)
- High area under curve values, not necessarily to get good grading performance (Teoh et al.^12^; Hafezi-Nejad et al.^13^)
- Difficulty in generalization ability due to region-specific machine learning approaches (Jamshidi et al.^11^)
- Requirement of large datasets (Tolpadi et al.^14^; Guan et al.^15^)
- Datasets possessing millions of distinct data points (Chen et al.^6^)
- More elapsed training and testing hours (Alexos et al.^10^; Jamshidi et al.^11^)
- No guarantee on scalability and reliability aspects (Yeoh et al.^16^; Wang et al.^17^)
- Black box nature of the machine learning models (Kokkotis et al.^18^; Mahesh et al.^24^)
- Scientific interpretation becomes complicated due to the increased number of layers (Chan et al.^25^)
- Lack of visualization of the diagnostic and detection models (Gan et al. ^26^)
- Transparency of the defined model is not maintained (Jamshidi et al.^30^)
- Models get limited by the applicability of the biases (Kijowski et al.^32^)
- Regulatory gap in the grading of the knee joint based images (Ibraheem et al.^33^)
- Difficulty in predicting the long-term outcomes of the models for total knee replacement due to osteoarthritis (Hu et al.^34^; Abd El-Ghany et al.^35^)
- Security aspects and adversarial attacks also not established (Mahum et al.^36^)
- Inherent biases of the clinical data resulting in reducing the accuracy level of grading (Teoh et al.^31^; Hu et al.^34^)

Table 1 presents the literature review of key contributions developed for this study in previous years. Under these circumstances, it is highly essential to develop and model suitable identification and classification models for the captured human knee joint images to detect the existence of osteoarthritis. The developed models are ensured with better stability and reliability and attains the most possible level of accuracy so that the patients shall be treated in advance and their pain gets relieved. Henceforth, the developed models should be a solution provider for this healthcare sector and thus in this research study, it is proposed to develop a novel hybrid optimization based deep learning model for detecting and classification grading of the captured knee joint images from X-ray devices. The proposed hybrid GAO-DCNN model combines the features of the Gaussian based Aquila optimization algorithm and the dual convolutional neural network model and identifies the osteoarthritis patients and helps in grading the knee OA. All the above limitations leading to disrupting the generalization ability and learning ability of the existing techniques are well handled with the modelled new Gaussian Aquila Optimizer based DCNN model for detecting and grading the osteoarthritis human knee images. With its optimal weight and bias coefficients, the proposed GAO based DCNN model will overcome the reliability and scalability issues observed in earlier works. Optimal coefficients intend to drive the model to attain better grading accuracy for the considered knee X-ray image datasets.

**Table 1:** Review on existing literature works.

References	Methodology Adopted	Limitations of the study
Tiulpin and Saarakkala^2^	Deep convolutional neural network model	Difficulty in feature extraction due to loss in capture of end points of images
Liu et al.^3^	Faster R-Convolutional neural network approach	Existence of inherent biases in the clinical data leading to delayed convergence
Chen et al.^4^	Ordinal loss based deep neural learning model	Low grading rate compared to KL grading technique
Teoh et al.^12^	Review on different machine learning techniques for OA classification	Higher Area Under Curve values, but grading performance not improved
Jamshidi et al.^11^	Individualized machine learning models	Difficulty in generalization ability due to region-specific techniques adopted
Guan et al.^15^	Deep learning risk assessment models with 21-layers	Model requires large number of datasets for validation
Alexos et al.^10^	Variants of machine learning approaches	More elapsed training and testing hours, resulting in increasing the computational burden
Yeoh et al.^16^	Basic Deep Learning Neural Network model with 11-layers defined including convolutional layers and pooling layers	No guarantee on scalability and reliability aspects. Premature convergence of the network model
Kokkotis et al.^18^	Fuzzy based feature selection and machine learning based OA classification	Difficulty in attaining the fuzzy rules and mapping them to get the most prominent features
Mahesh et al.^24^	Adaboost ensemble methods	Black box nature of the machine learning models resulting in poor error minimization rate
Chan et al.^25^	Machine learning models to decipher multi-etiology of OA	Increased number of layers resulting in complicated scientific interpretation
Teoh et al.^31^	Multitask deep hybrid learning approach	Increased computational complexity of the multitask model
Kijowski et al.^32^	Deep learning models—CNN with required no. of layers	Models get limited by the applicability of the biases
Ibraheem et al.^33^	Enhanced sequential deep learning technique	Regulatory gap in the grading of the knee joint images
Abd El-Ghany et al.^35^	Fine-tuned deep learning model	Difficulty in predicting the long-term outcomes of the models
Mahum et al.^36^	Deep Learning CNN Model	Complex fully connected dense network and elapses more computational time
Hu et al.^34^	DeepKOA technique	Complex structure of DeepKOA and no generalization ability ascertained

On the other hand, the limitations of the existing methods include,

- Insensitive to the changes occurring in the patients
- KL system considers a linear progression of radiograph images
- Do not consider the joint space of the lateral and medial compartments
- Inconsistency of the original description
- Difficulty in identifying the definite osteophytes
- No correlation to arthroscopic evidences
- Difficulty in discriminating Joint space narrowing
- Not able to diagnose osteophyte occurrences
- Lack of recognition of distinct arthritis

Due to which, the developed models should be a solution provider for this healthcare sector and thus in this research study, it is proposed to develop a novel hybrid optimization based deep learning model for detecting and classification grading of the captured knee joint images from X-ray devices.

## Proposed methodology

In this section of the paper, the proposed methodology of Gaussian Aquila Optimizer based Dual Convolutional Neural Network is modelled. The mathematical equations employed for proposing the model, the flow of the algorithmic process and the hybridization process of the GAO optimizer and the new Dual CNN model has been elucidated. For substantiating the validity and effectiveness of the developed detector and OA classifier, it is tested on the datasets pertaining to the humans captured from the X-ray devices. For training, testing and validating the developed novel hybrid GAO-DCNN model, the X-ray images of Knee Joints of humans were utilized. The Knee Joint X-ray images used in this research study for simulation purpose is collected from Sri Nalam Ortho Clinic, Dindigul District, India with necessary ethics approval from the hospital organization. Based on the received ortho images, the dataset to be employed is constructed. The complete dataset after careful examination is split into normal knee image dataset and osteoarthritis knee image dataset, so as to be used for initiating the deep learning training process. The normal knee image dataset comprises of 1267 normal knee images and the osteoarthritis knee image dataset is built with 1016 osteoarthritis knee images.

**Figure 2:**
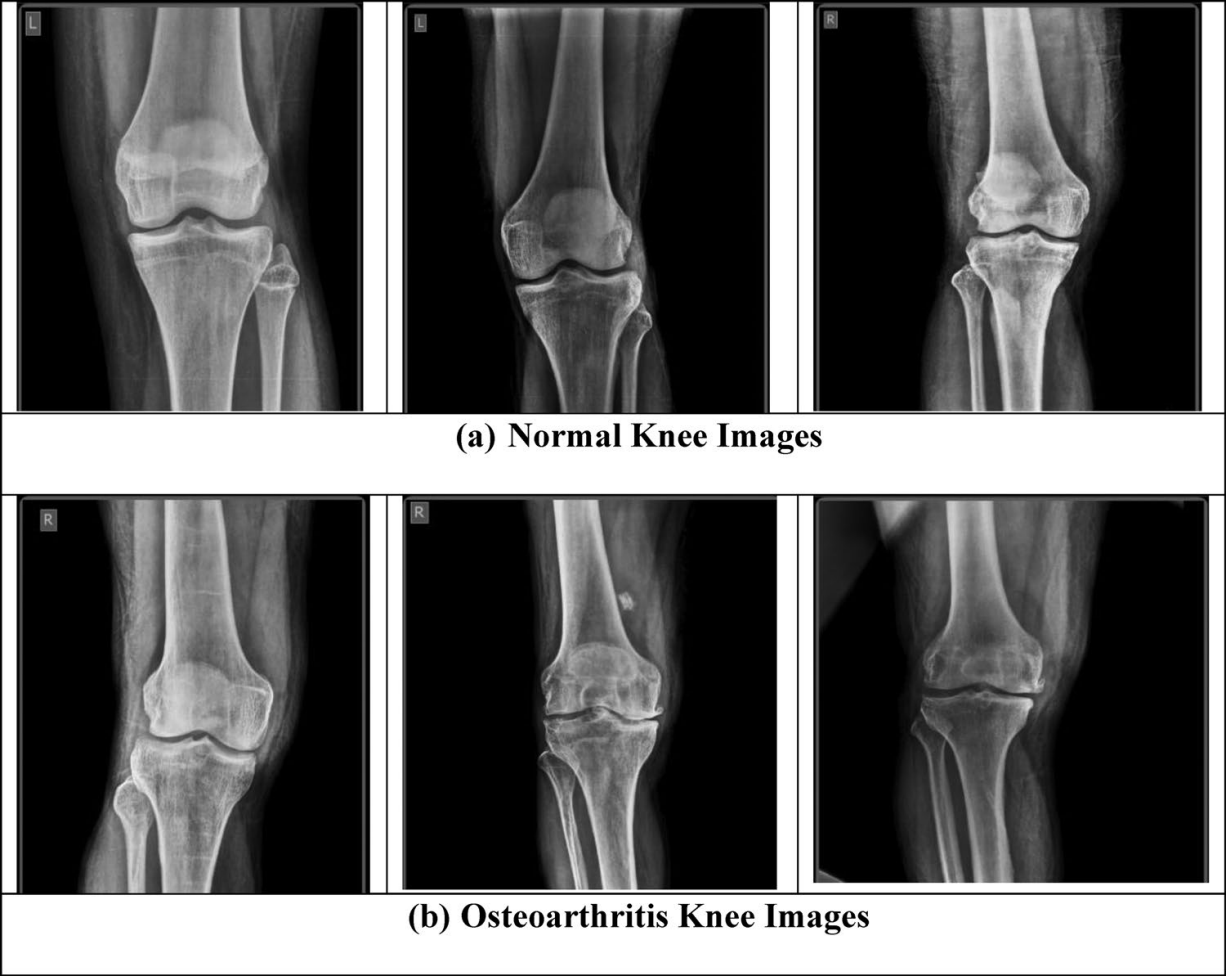
Constructed Sample Dataset for Normal and Abnormal Knee images. Source: Sri Nalam Ortho Clinic.

**Figure 3:**
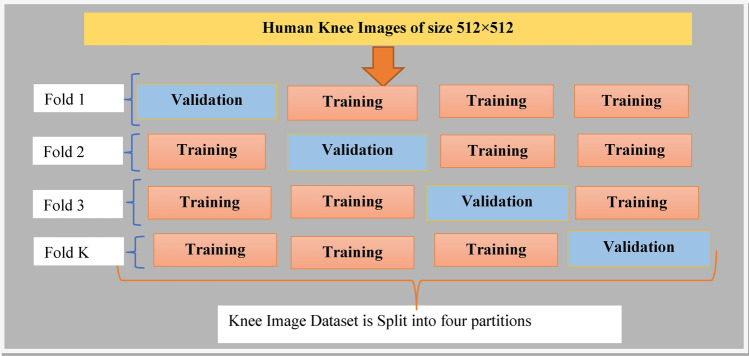
k-Fold Cross validation applied for Knee joint images.

The captured knee joint images belong to persons within the age group of 15 years and 70 years irrespective of gender types. The size of each knee images for both normal and osteoarthritis case pertains to 512 × 512 pixels specifying the image width and height respectively. Figure 2 presents the sample of the normal knee images and osteoarthritis knee images as received from the Ortho Clinic and are to be used in the developed proposed optimized deep learning model. It is well noted from the Fig. 2, the difference among the normal knee and osteoarthritis affected knee based on the undulation observed. In this work, k-fold cross validation is applied for the image datasets and is as shown in Fig. 3. Based on the cross-validation done, the images are fed into the proposed hybrid GAO-DCNN learning model for training, testing and validation to maintain balance of the datasets when presented for learning.

In this study, osteoarthritis knee abnormality is detected and graded using the human X-ray images with an optimized dual CNN architectural model. At the training stage of designed DCNN classification module, the normal knee images and osteoarthritis knee images are enhanced individually and knee joints in both normal and abnormal images gets segmented. Subsequently, the morphological features get computed from the knee joint regions of both normal and abnormal images. Then these morphological features are trained with the new Dual CNN architecture, which computes the Trained Matrix (TM). At the testing phase of new DCNN classifier, the knee images of the same patient at different orientation are captured, segmented and classified with respect to the computed trained matrix attained from the trained DCNN classifier. This mechanism classifies the source knee image into either normal or osteoarthritis knee image. The developed model facilitates in detecting the existence of osteoarthritis at an early stage and make the individuals to be free from pain or surgery.

### Classic Aquila optimizer—revisited

Inspired by the natural behaviour of the bird Aquila on catching its prey formulated the Aquila Optimization Algorithm (AQO) and presently for the past two years used widely for solving engineering and scientific application-based problems^37–40^. In this research work, AQO algorithm is chosen among all the existing various bio-inspired, nature-inspired, physics and human inspired, and all other population-based stochastic optimization algorithms due to the following factors,

- Diversified operation of exploration and exploitation in narrowed and expanded form
- Ease of computation towards optimal solution attainment
- Distribution of bird movements is even
- Operates with minimal number of parameters
- Possess higher stochastic ability
- Expanded and Narrowed Process makes the search progress better

With the above merits of the AQO, in this research study a modified version of AQO is developed and applied for the identification and classification of osteoarthritis in humans. The mechanisms followed by bird Aquila to catch hold of the prey includes—high soar with vertical stoop, contour flight with short glide, low fly with a gradual descent and at last walk and grab the prey. Using this behaviour of Aquila for grabbing the prey, the optimization algorithm is modelled considering each stage of the bird hunt. The algorithmic steps of AQO for attaining optimal solutions include initialization, expanded and narrowed exploration, expanded and narrowed exploitation. The basic algorithm of AQO is as given below:

**Table:** 

*Initialization:*	Required parameters are initialized for the run of the algorithm. Initial populations get randomly generated to start the AQO process flow
*Expanded Exploration:*	The search space starts exploring from the high ascend, performing the high soar with vertical stoop and is given by, $S_{1} \left( {j + 1} \right) = S_{best} \left( j \right) \times \left( {1 - \frac{j}{J}} \right) + \left( {S_{A} \left( j \right) - S_{best} \left( j \right) \times rand} \right)\quad (1)$ where, *S*_*1*_*(q* + *1)* is the solution of the forthcoming iteration ‘*j*’ and this pertains to get computed with initial search approach *S*_*1*_. *S*_*best*_*(j)* is the best solution determined among all the iterations. The exploration mechanism operates with control factor $\left(1-\frac{j}{J}\right)$ and ‘J’ denotes the maximum number of iterations to be elapsed and ‘*j*’ specifies the current iteration. *S*_*A*_*(j)* in the location mean component of the present solution and ‘*rand*’ is taken from 0 to 1. ‘*S*_*A*_’ is computed with, $S_{A} \left( j \right) = \frac{1}{M}\mathop \sum \limits_{i = 1,k}^{M} S_{ik} \left( j \right), for\, all\, k = 1,2, \ldots ..dimension\quad (2)$ In Eq. (2), ‘*M*’ is the population size
*Narrowed Exploration*:	This step performs the contour flight with short glide strategy of Aquila wherein bird gets encircled around the prey for attacking. This mechanism is specified with, $S_{2} \left( {j + 1} \right) = S_{best} \left( j \right) \times levy\left( {Q_{s} } \right) + \left( {S_{rd} \left( j \right) + \left( {a - b} \right) \times rand} \right)\quad (3)$ Where, ‘*S*_*2*_*(j* + *1)*’ gives the solution of forthcoming iteration ‘*j*’, *S*_*rd*_*(j)* indicates the random solution of [1, J], *S*_*best*_*(j)* gives the best solution pertaining to that iteration and ‘*rand*’ generates number from 0 to 1 and ‘*a*’ and ‘*b*’ corresponds to spiral search shape and is given by, $a = l \times sin\left( \phi \right)\quad (4)$ $b = l \times \cos \left( \phi \right)\quad (5)$ ‘*l*’ denotes the radius in respect of spiral shape and ‘ϕ’ is the angle movement. Levy flight is obtained with, $levy\left( {Q_{s} } \right) = r \times \frac{l \times \beta }{{\left| x \right|^{{\frac{1}{\alpha }}} }}\quad (6)$ Where, *Q*_*s*_ indicates the dimension space of the levy flight distribution, *r* is constant value of 0.1, *l* and *x* are random numbers from 0 to 1, *α* is 0.5 and ‘*β*’ is indicates the parameter of levy flight distribution
*Expanded Exploitation*:	Following the mechanism of Aquila, low flight with a slow descent attack, the third strategy is carried out to plunge the targeted space and move closer towards the prey so as to attack. Equation (7) specifies the operation of this strategy, $$\begin{aligned} S_{3} \left( {j + 1} \right) = & \left( {S_{best} \left( j \right) \times S_{A} \left( j \right)} \right) \times \left( { - rand} \right) \\ & + \left( {\left( {UB - LB} \right) \times rand + LB} \right) \times \mu \quad (7) \\ \end{aligned}$$ Where, the upper bound and lower bound are given by *UB* and *LB*, λ and μ indicates the exploitation tuning factors
*Narrowed Exploitation*:	This phase implments the walk and grab attack strategy at the last step of Aquila prey catching mechanism and known as the narrowed exploitation process and is written using equation, $$\begin{aligned} S_{4} \left( {j + 1} \right) = & \in \times S_{best} \left( j \right) - \left( {H_{1} \times S\left( j \right) \times rand} \right) \\ & - \left( {H_{2} \times levy\left( {Q_{s} } \right) + rand \times H_{2} } \right)\quad \quad (8) \\ \end{aligned}$$ In above equation, $\in$ denotes the quality function that balances the search process, the movement *H*_*1*_ and slope *H*_*2*_ are evaluated with, $\in \left( j \right) = j^{{\frac{2 \times rand - 1}{{\left( {1 - j} \right)^{2} }}}} \quad (9)$ $H_{1} = 2 \times rand - 1\quad (10)$ $H_{2} = 2 \times \left( {1 - \frac{j}{J}} \right)\quad (11)$
*Termination Criterion:*	The process gets terminated until the required minimized/ maximized value of the fitness function is attained or the maximum number of generations are met

The basic Aquila Optimizer do not employ mutation operation. The proposed new Gaussian Aquila Optimizer employs Gaussian based Mutation at the end after all the four steps of the algorithm. The applicability of Gaussian mutation plays a vital role in getting diversified solution i.e., a new solution from search space which is not obtained from regular search mechanism. Gaussian mutation involves the search process at the exploitation phase only within the global best values, thereby attains the most suitable optimal values and also no delayed or premature convergence is encountered. Actually, in the proposed GAO, the operations include,

- Expanded Exploration
- Introduction of new Momentum Control Factor (MCF)
- Improved Narrowed Exploration
- Improved Expanded Exploitation
- Narrowed Exploitation
- Gaussian Mutation

The generational cycle to be elapsed will be controlled by the momentum control factor which is not in the case of basic Aquila Optimizer model.

### Novel Gaussian Aquila optimizer

The exploration mechanism is done in two phases of narrowed exploration and expanded exploration in the classic AQO algorithm. The modelled new Gaussian Aquila Optimizer employs Gaussian mutation so as to increase the exploration ability in the search space i.e., the applicability of the non-uniform mutation strategy improves the exploration phase. Additionally, in the new GAO algorithm a momentum control factor (MCF) gets introduced to handle the weak notion of the narrowed exploration and expanded exploitation process value and this MCF intends to reduce the elapsed number of generations for attainment of the optimal solution. The proposed GAO algorithm is developed as:

*Momentum Control Factor (MCF)*: The introduced momentum control factor acts to control the generation cycle elapsed and also the step size at the time of basic search mechanism including the position and motion of Aquila traversal. On generation cycle execution, the Aquila movement is reduced steadily and thereby the accuracy in the search mechanism gets increased. As a result, the absolute value of the MCF decreases the progressive generational cycle and is defined as,

12 $$MCF(j)={M}_{o}.exp\left(1-\frac{j}{J}\right)\times {N}_{s}$$

13 $${N}_{s}=\left\{\begin{array}{c}1, if\, r<0.6\\ -1, Otherwise\end{array}\right.$$

In the above eqns. (12) and (13), ‘*M*_*o*_’ specifies the position constant greater than zero and is set to 1.2 after trial runs. The factor $\left(1-\frac{j}{J}\right)$ controls the bird movement over the number of generations, ‘*r*’ indicates a random number between 0 to 1, and ‘*N*_*s*_’ denotes the momentum control parameter using which is the direction and movement of Aquila is controlled.

*Improved Narrowed Exploration Phase*: In the proposed GAO algorithm, the narrowed exploration process of the regular AQ algorithm gets modified by adding the *MCF(j)* and this improvises the search mechanism for a complete search process with the short glide attack. Here, the bird intends to search the complete solution space for varied directions and speed, then gets encircled to attack the prey.

14 $${S}_{2n}\left(j+1\right)={(S}_{best}\left(j\right)-{S}_{present}(j))\times MCF\left(j\right)+\left({S}_{rd}\left(j\right)+\left(a-b\right)\times rand\right)$$

Equation (14) indicates an improved version by adding the momentum control factor using which the full positional search is carried out and the current Aquila position is considered for computing the global best of this step. In Eq. (3), ‘*S*_*2n*_*(j* + *1)*’ pertains to the solution of next iteration ‘*j*’, *S*_*rd*_*(j)* specifies the random solution of [1, J], *S*_*best*_*(j)* corresponds to the best solution and ‘*a*’ and ‘*b*’ is spiral shape corresponding to search (14), *S*_*present*_*(j)* denotes the present position and *MCF(j)* represents new operator in Eq. (12).

*Improved Expanded Exploitation Phase*: At the third phase, the bird enhances its search process and searches completely around the prey and attacks it. Takes a complete flight followed by a descent attack and then subsequently plunging and encircling the prey and attacking it. This improved Aquila action is given by,

15 $$\begin{aligned} S_{3n} \left( {j + 1} \right) = & \left( {(S_{best} \left( j \right) - S_{present} \left( j \right)) \times S_{A} \left( j \right) \times MCF\left( j \right).\left( {1 - \frac{j}{J}} \right)} \right) \times \left( { \lambda - rand} \right) \\ & + \left( {\left( {UB - LB} \right) \times rand + LB} \right) \times \mu \\ \end{aligned}$$

In the above eqn., ‘*S*_*3n*_*(j* + *1)*’ indicates the solution to be used for the next iteration, ‘*S*_*present*_*(j)*’ denotes the current position of Aquila, *S*_*N*_*(j)* indicates the mean value as calculated using Eq. (2), *UB* and *LB* denotes the upper bound and lower bound, ‘*MCF(j)*’ evaluated with Eq. (12) and controls the bird movement at the time of search.

*Gaussian Mutation (GM)*: Basically, mutation process operates effectively in the exploitation phase and this Gaussian mutation gets introduced in the new GAO algorithm and thereby overcoming local optima trap. Gaussian mutation is indicated mathematically as,

16 $${S}_{GlBest}(j)=S(j)\times \left(1+{S}_{gaussian}(j)\right)$$

In Eq. (16), ‘*S*_*GlBest*_*(j)*’ denotes the mutated positions so as to reach the global positions, ‘*S*_*gaussian*_*(j)*’ indicates the Gaussian mutation carried out with,

17 $${S}_{gaussian}(j)={e}^{-{{S}_{present}}^{2}}$$

Equation (17) defines the Gaussian operator used to improve the exploitation mechanism through its normal distribution operation. Equation (17) allows the mutation operator to search the solution space in a uniform manner at the initial phases and then locally in subsequent phases. This avoids the random selection and improves the probability of new population getting generated overcoming the local and global optimal trapping. Table 2 presents the pseudocode for the proposed new Gaussian Aquila Optimizer algorithm.

**Table 2:**
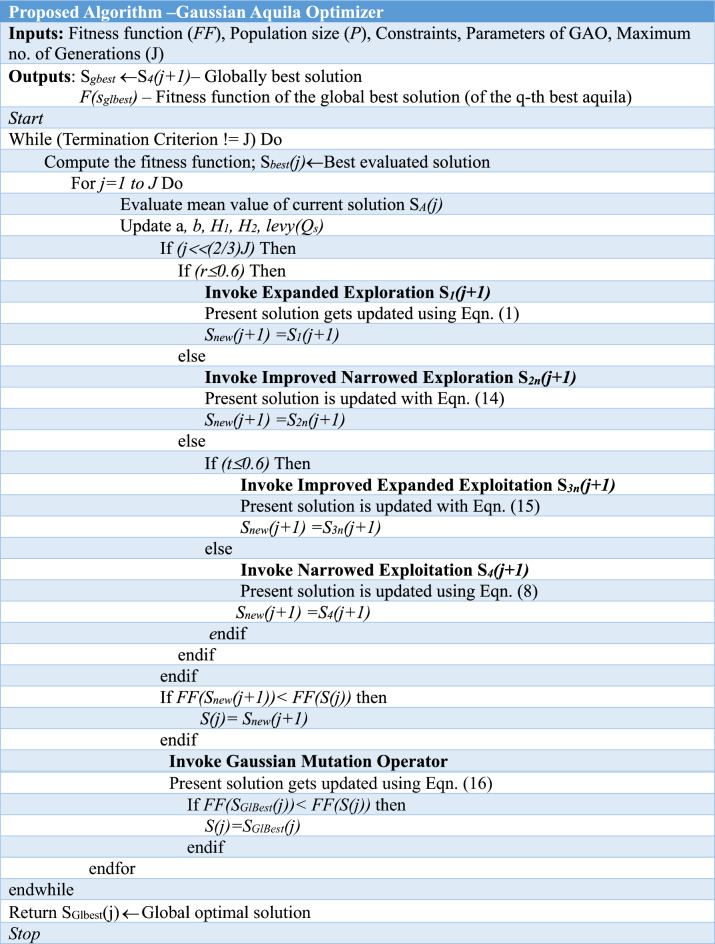
Pseudocode of new Gaussian Aquila optimizer algorithm.

### Modelled dual convolutional neural network

**Figure 4:**
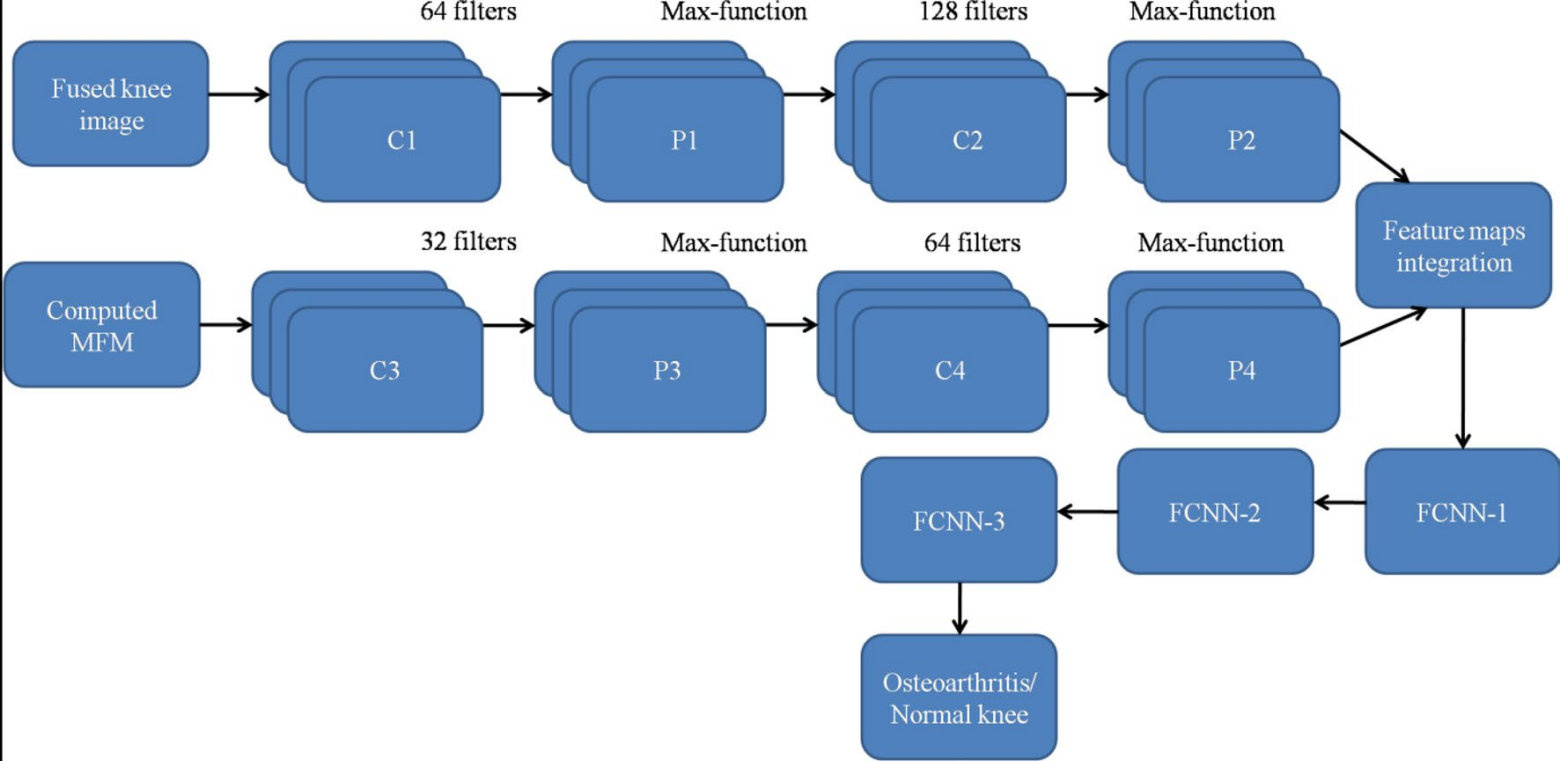
Proposed stack Dual CNN model as Osteoarthritis classifier.

In this research study, Dual CNN (DCNN) model has been proposed to detect the osteoarthritis diseases in knee images and the fully connected layer weights are tuned for its optimal values using the novel Gaussian Aquila Optimizer. The first convolutional neural network is structured using two independent Convolutional layers (C1 and C2) and two pooling layers (P1 and P2) as shown in Fig. 4. The C1 layer consists of 64 set of filters and C2 layer consist of 128 filters. This first CNN module obtains fused knee image as input and this image is passed through the set of C1 and C2 layers to produce the feature maps-1. At the end of each Convolutional layer, pooling layer (which incorporates Max-function) is placed to shrink the output sequences from the Convolutional layer.

The second CNN of the Dual CNN is structured using two independent Convolutional layers (C3 and C4) and two pooling layers (P3 and P4) as shown in Fig. 4. The C3 layer consists of 32 set of filters and C4 layer consist of 64 filters. This second CNN module obtains Morphological Feature Matrix (MFM) as input and this MFM is passed through the set of C3 and C4 layers to produce the feature maps-2. At the end of each Convolutional layer, pooling layer (which incorporates Max-function) is placed to shrink the output sequences from the Convolutional layer. Then, these two feature maps such as feature map-1 and feature map-2 are integrated to produce the final feature map. This feature map is passed through the three Fully Connected Neural Networks (FCNN) to produce the knee image classification responses as either Osteoarthritis or normal.

In training phase, the enhanced images are directly given to the segmentation process and here learning is enabled to get trained with these images. Deep learning is carried out in such a way in training process, such that with the enhanced images itself, the maximum number of morphological features are captured and the learning progresses with the defined activation functions. In training even with minimum features, the network is capable of learning and to attain convergence and thereby to derive the Trained Matrix for use at testing phase.

**Figure 5:**
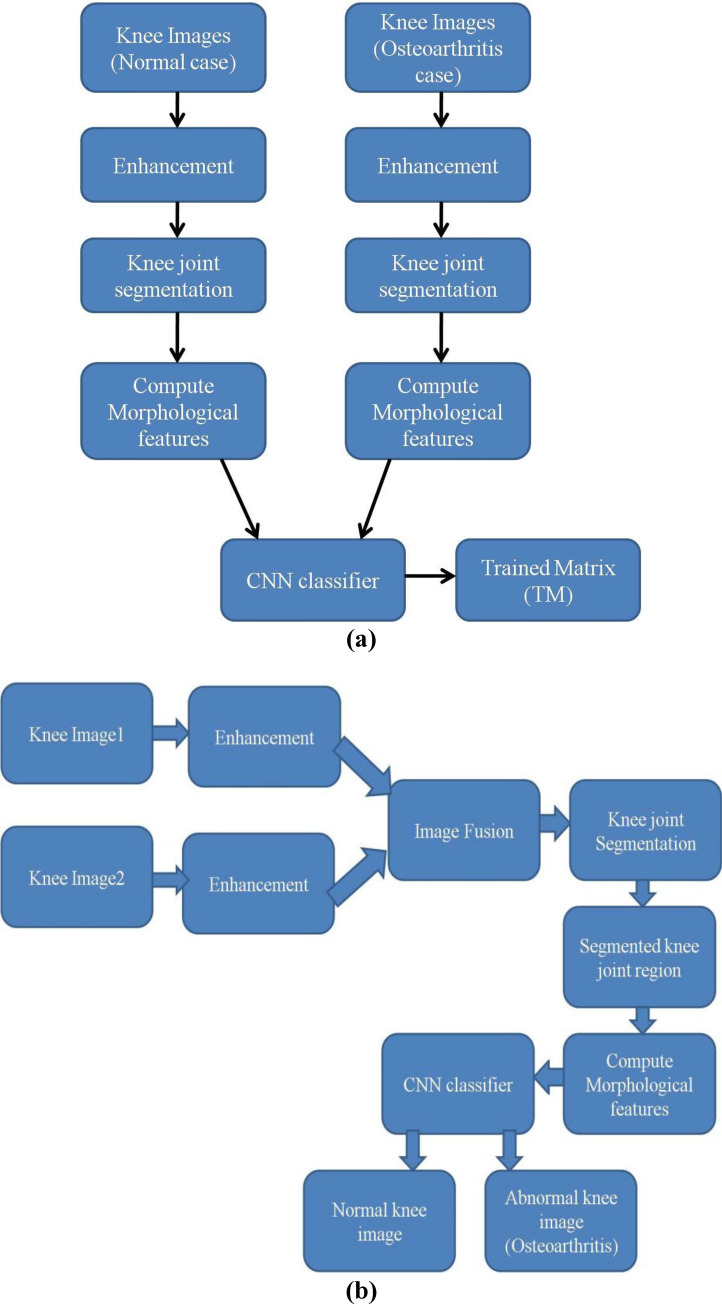
Proposed Dual CNN Operational Flowchart (**a**) Training phase (**b**) Testing phase.

**Figure 6:**
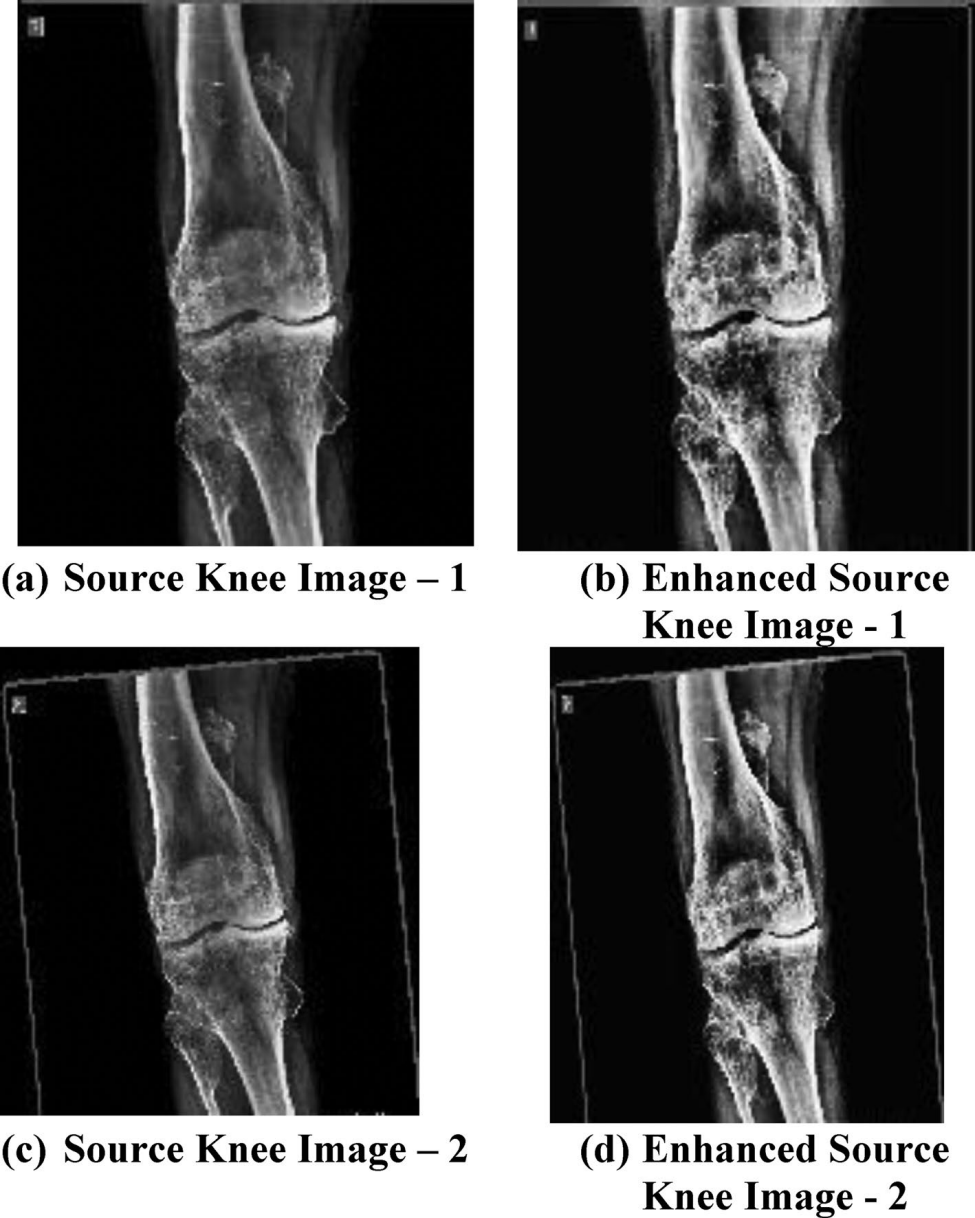
Source knee image and its enhanced version for sample datasets.

On the other hand, in case of testing phase, no features should be lost i.e., the features that exist at the edges should also be not lost to get a better testing accuracy. The edges become clearly visible in the enhanced knee images. These edge pixels are used to locate the knee joints. Therefore, it is highly essential to fuse these two enhanced images to locate the knee joint regions more accurately. The enhanced knee images are now fused into unique knee image using wavelet transform in this research study. Figure 5 presents the Dual CNN training phase of Osteoarthritis detection and Fig. 6 presents the Dual CNN testing phase for giving raw testing knee image inputs and using the trained matrix (TM) coefficients to evolve the testing results.

#### Image enhancement

The X-ray images employed in this research study possess low resolution intensity values. Due to which, it is highly difficult to detect any abnormality in X-ray images. In order to improve the pixel intensity and resolution to the higher level of intensity values, Adaptive Local Histogram Equalization (ALHE) method is used for enhancing the pixel values in X-ray images. Even though numerous enhancement methods available, ALHE method is used in this study due to its edge pixel restoration property during enhancement process. The algorithmic steps adopted for this image enhancement process is as given below:

**Table:** 

*Pseudo Algorithm: Image enhancement*	
*Inputs:*	Human Knee Images Captured from X-Ray devices
*Outputs:*	High intensity enhanced knee images
*Start*	
*Step 1:*	Complete knee image datasets are split into ‘*N*’ number of non-overlapping regions and each non-overlapping region is having the size of 5 × 5 pixels as image width and height respectively
*Step 2:*	Compute histogram count of each non-overlapping region
*Step 3:*	Evaluate Cumulative Distributive Function (CDF) using histogram count in each non-overlapping region
*Step 4:*	Multiply the computed CDF value with each pixel individually in each non-overlapping regions to enhance the pixel intensity, which produces the enhanced knee image
*Stop*	

#### New knee image fusion

From the previous step of the proposed algorithm, the edges get clearly visible in the enhanced knee images. These edge pixels are used to locate the knee joints. Therefore, it is necessary to fuse these two enhanced images to locate the knee joint regions more accurately. The enhanced knee images are now fused into unique knee image using wavelet transform in this research study. This image fusion algorithm is as given below:

**Table:** 

Pseudo Algorithm: Knee Image Fusion	
Inputs:	Enhanced Source Knee Image-1 and Enhanced Source Knee Image-2
Output:	Fused Knee Image
Start	
Step 1:	The enhanced knee images are converted into luminance and chrominance pattern formats using the following equations $\left[ {y1;cb1;c1r} \right] = rgb2ycbcr \,\left( {E1} \right)\quad (18)$ $\left[ {y2;cb2;c2r} \right] = rgb2ycbcr \,\left( {E2} \right)\quad (19)$ Whereas, E1 and E2 are the enhanced knee images and $y1$ and $y2$ are the luminance pattern formats of enhanced knee images respectively
Step 2:	The luminance knee images are decomposed into low and high frequency sub bands using Daubachies-4 symmetric mode two-dimensional DWT using the following equations $\left[ {A1;H1;V1;D1} \right] = dwt \,\left( {y1, \,db4, \,^{\prime}sym^{\prime}} \right)\quad (20)$ $\left[ {A2;H2;V2;D2} \right] = dwt \,\left( {y2, \,db4, \,^{\prime}sym^{\prime}} \right)\quad (21)$ In Eqs. (20) & (21), low and high frequency sub bands of decomposed enhanced knee image 1 are denoted as $A1 \;and\; H1,V1,D1$ respectively and low and high frequency sub bands of decomposed enhanced knee image 2 are denoted as $A2 and H2,V2,D2$ respectively
Step 3:	Compute the Eigen values of high pass sub bands and also compute maximum of these computed Eigen values using the following equations $Ei1 = Eigen \,\left( {A1} \right)\quad (22)$ $Ei2 = Eigen \,\left( {A2} \right)\quad (23)$ $Max - Ei = Maximum \,\left( {Ei1,Ei2} \right)\quad (24)$
Step 4:	Compute the Eigen values of low pass sub bands and also compute maximum of these computed Eigen values using the following equations $\left[ {{\text{Ei}}_{{{\text{h}}1}} ;{\text{Ei}}_{{{\text{v}}1}} ;{\text{ Ei}}_{{{\text{d}}1}} } \right] = {\text{Eigen }}\left( {{\text{H}}1;{\text{V}}1;{\text{D}}1} \right)\quad (25)$ $\left[ {{\text{Ei}}_{{{\text{h}}2}} ;{\text{Ei}}_{{{\text{v}}2}} ;{\text{ Ei}}_{{{\text{d}}2}} } \right] = {\text{Eigen }}\left( {{\text{H}}2;{\text{V}}2;{\text{D}}2} \right)\quad (26)$ $Max - Elow1 = Maximum \,\left( {{\text{Ei}}_{{{\text{h}}1}} ;{\text{Ei}}_{{{\text{v}}1}} ;{\text{ Ei}}_{{{\text{d}}1}} } \right)\quad (27)$ $Max - Elow2 = Maximum \,\left( {{\text{Ei}}_{{{\text{h}}2}} ;{\text{Ei}}_{{{\text{v}}2}} ;{\text{ Ei}}_{{{\text{d}}2}} } \right)\quad (28)$ $Max - Elow = Maximum \,\left( {Max - Elow1,Max - Elow2} \right)\quad (29)$
Step 5:	Multiply the decomposed coefficient of high pass sub band of both enhanced images with $$Max - Ei$$ using the following equations $$\left[ {A1 - new;\,A2 - new} \right] = \left[ {A1* Max - Ei; \,A2* Max - Ei} \right]\quad (30)$$ In Eq. (30), $$A1$$$$- new$$$$- new$$$$- new$$$$- new$$$$- new$$$$- new$$$$- new- new- new- new$$ and $$A2 - new$$ are the newly decomposed high pass sub bands of enhanced image 1 and enhanced image 2, respectively
Step 6:	Multiply the decomposed coefficient of low pass sub band of both enhanced images with $$Max - Ei$$ using the following equations $$\left[ {H1 - new;\,V1 - new;\,D1 - new} \right] = \left[ {H1* Max - Elow; \,V1* Max - Elow; \,D1* Max - Elow} \right]\quad (31)$$ $$\left[ {H2 - new;\,V2 - new;\,D2 - new} \right] = \left[ {H2* Max - Elow; \,V2* Max - Elow; \,D2* Max - Elow} \right]\quad (32)$$ In Eq. (31) & (32), $$H1$$$$- new, \,V1 - new$$$$- new, \,V1 - new$$$$- new, \,V1 - new$$$$- new, \,V1 - new$$$$- new, \,V1 - new$$$$- new, \,V1 - new$$$$- new, \,V1 - new- new, \,V1 - new- new, \,V1 - new- new, \,V1 - new$$ and $$D1 - new$$ are the newly decomposed low pass sub bands of enhanced image 1, respectively,$$H2 - new, \,V2 - new$$ and $$D2 - new$$ are the newly decomposed low pass sub bands of enhanced image 2, respectively
Step 7:	Perform Inverse DWT (IDWT) using the newly reconstructed low and high pass sub bands using the following equations $\left[ A \right] = A1 - new + A2 - new\quad (33)$ $\left[ {H,V,D} \right] = \left[ { H1 - new + H2 - new; \,V1 - new + V2 - new; \,D1 - new + D2 - new} \right]\quad (34)$ $Fused knee image = idwt\left( {A,H,V,D} \right]\quad (35)$
Stop	

#### New knee joint segmentation

The segmentation of knee joint over the fused knee images plays an important role in knee image classification system for osteoarthritis detection. The connected component labelling algorithm is proposed in this research study to segment the knee joint region in the fused knee image. This algorithm converts the grey scale image into binary image and places 3 × 3 mask window on the binary fused knee image. Further, it computes the number of black and white pixels in this 3 × 3 mask window. If the number of black pixel count is high, then change all pixels into black pixels such as ‘0’. If the number of white pixel count is high, then change all pixels into white pixels such as ‘1’. Then move this sub window to the next overlapping blocks and repeat the same procedure till the end of the fused image. Employing this technique, the segmentation is carried out for the fused images and then classification mechanism is done for the knee images.

### Proposed hybrid GAO-DCNN learning model

In this research study, the proposed improved Gaussian Aquila Optimizer algorithm combined with the deep dual convolutional neural network is employed as detector and classifier for the knee images for classifying normal and osteoarthritis. The new GAO with its Gaussian mutation improves the narrowed and expanded exploitation mechanism and it results in attaining the accurate solutions. A complete balance of exploration and exploitation is obtained with the GAO and is used to find the weight values for the fully connected layer of the Dual convolutional neural network. The algorithm for the new hybrid GAO-DCNN learning model is as given below in Table 3.

**Table 3:**
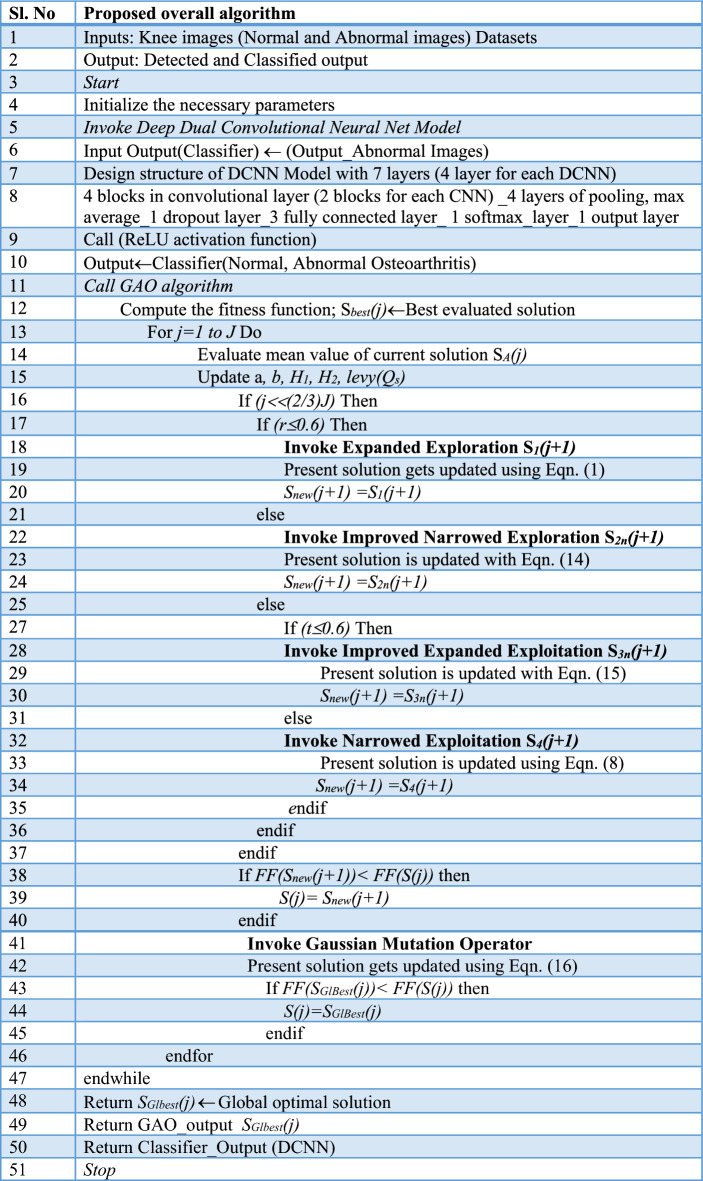
Developed GAO-DCNN algorithm for Osteoarthritis classification.

New hybrid GAO-DCNN algorithm developed in this research study is used for detection and classification of the normal and osteoarthritis images of the captured human knee images and the performance metrics are evaluated to prove the efficacy of the proposed model.

### Ethics approval and informed consent to participate

Necessary ethical standard was maintained in this research study. All methods were carried out in accordance with relevant guidelines and regulations of Medical Council of India. All experimental protocols were approved by Sri Nalam Ortho Clinic, Dindigul. Consent was obtained from all subjects with the approval from Sri Nalam Ortho Clinic Authorities. “Written Informed Consent” has been obtained from participants for whom knee X-ray image datasets were attained. The methods were carried out based on the norms and guidelines of Medical Council of India (MCI). Board of Council members (BCM) of the Sri Nalam Ortho Clinic is the ethical committee that approved the study.

## Results and discussions

In this research study, the modelled new hybrid GAO-Dual CNN model is simulated and tested for its superiority for the identification and classification of osteoarthritis for the human knee images. Simulation was carried out in Intel Core i7‑11390H 5 GHz processors with 10 GB RAM and 1 TB hard disk drive in MATLAB R2023a version. The knee images pertaining to different humans obtained from the Sri Nalam Ortho Clinic are constructed to form the datasets. The ethical committee that approved this study and the collection of knee joint X-ray image datasets of humans is the Board of Council Members (BCM) of Sri Nalam Ortho Clinic, Dindigul. The complete dataset was split into normal knee image dataset and osteoarthritis knee image dataset. 1267 normal knee images and 1016 osteoarthritis knee image dataset was used for training and testing the developed hybrid GAO-DCNN learning model. In case of both, normal and osteoarthritis knee images, the size of the image corresponds to 512 × 512-pixel width and height respectively. The morphological features are used to differentiate the morphological behaviour of each pixel in the segmented knee joint region. In this study, the morphological features mean, kurtosis, skewness, eccentricity, major axis length and minor axis length, are computed from the segmented knee joint region. These computed morphological features are integrated into Morphological Feature Matrix (MFM) and are fed into the hybrid GAO-DCNN learning model for detection and classification grading of osteoarthritis affected knee images.

The conventional CNN architecture requires higher number of training images for achieving significant classification results. The proposed novel DCNN architecture requires lower number of training images due to its dual CNN layer properties, which generates optimum number of features for producing significant classification results. Hence, in this research study, 20% of knee images in both cases are used for testing the proposed DCNN module and the remaining 80% of knee images in both cases are used for training the proposed DCNN module and subsequently k-fold cross validation is adopted. Out of all k-fold cross validation, as 80% training and 20% testing was observed to be versatile in all iterations, the final results were computed using this percentage of data. Hence, 253 normal knee images are used for testing and 1014 normal knee images are used for training. Henceforth, 203 Osteoarthritis knee images are used for testing and 813 Osteoarthritis knee images are used for training. The specifications of each internal modules of the proposed DCNN structure are listed in Table 4 and the values of the set parameters of GAO algorithm is given in Table 5.

**Table 4:** Layer design of the proposed DCNN model.

Modelled deep learning neural network	Layers designed	Number of filters	Layer kernel size	Number of neurons in each layer
CNN-1	C1	64	3 × 3	–
	P1	64	2 × 2	–
	C2	128	5 × 5	–
	P2	128	2 × 2	–
CNN-2	C3	32	5 × 5	–
	P3	32	2 × 2	–
	C4	64	7 × 7	–
	P4	64	2 × 2	–
DCNN	FCNN-1		–	4096
	FCNN-2		–	2048
	FCNN-3		–	2

**Table 5:** Parametric values of the proposed Gaussian aquila optimizer.

Parameters	Set values for GAO algorithm
No. of populations of Aquila	60
*λ*	0.1
*μ*	0.1
*H1*	[− 1, 1]
*H2*	[2, 0]
Mutation	Gaussian
Convergence criterion	10^–6^
No. of trial runs	32
Max iterations	Till the convergence criterion

With minimal number of images, the proposed GAO-DCNN achieves better grading rates for the osteoarthritis identified patients. Fivefold cross validation was applied for splitting the data for training and testing (Based on the cross validation, it will be 90:10, 80:20, 70:30, 60:40, 50:50) was initially done. Then during learning it was observed that with 80:20, it was versatile and the solutions were better, hence this percentage of split was further maintained. The advantages of Dual CNN layer lie in their feature extraction including the edge features without any loss of information. Each of the layer in Dual CNN performs the convolution, filtering, feature extraction, pooling and other dense layer operations to its best possible level and has attained better accuracy and grading rate compared to other models.

Normal Knee Detection Rate (NKDR) is the parameter metric which is defined as the ratio between the correctly detected normal knee X-ray images using the proposed DCNN and the total normal knee X-ray images. Osteoarthritis Knee Detection Rate (OKDR) is the parameter metric which is defined as the ratio between the correctly detected Osteoarthritis knee X-ray images by the proposed DCNN and the total Osteoarthritis knee X-ray images.

The source image and enhanced version of the considered source image is as shown in Fig. 6. Figure 6 is given for the sample datasets. Figure 6a shows the source knee image-1 and Fig. 6b shows its enhanced image; Fig. 6c shows the source knee image-2 (at 30° angle of inclination) and Fig. 6d shows its enhanced knee image. The varaitions of pixel intensities in knee joint region can be more significantly identified by taking the X-ray images at different angles. Therefore, 0° and 30° Field of View (FoV) are set for capturing the knee images.

**Figure 7:**
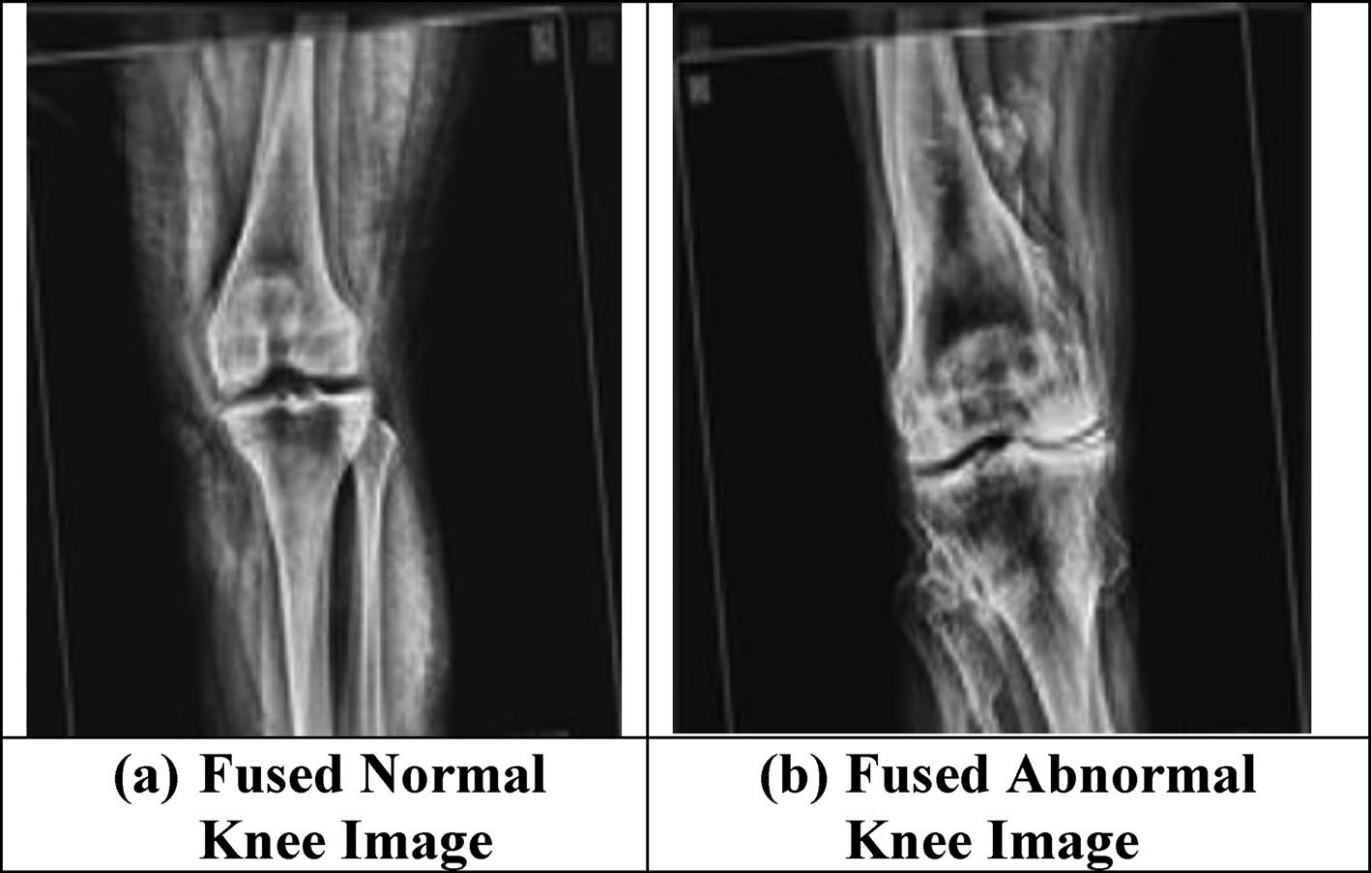
Fused images of normal and abnormal knee images. (Image Fusion Technique Applied).

**Figure 8:**
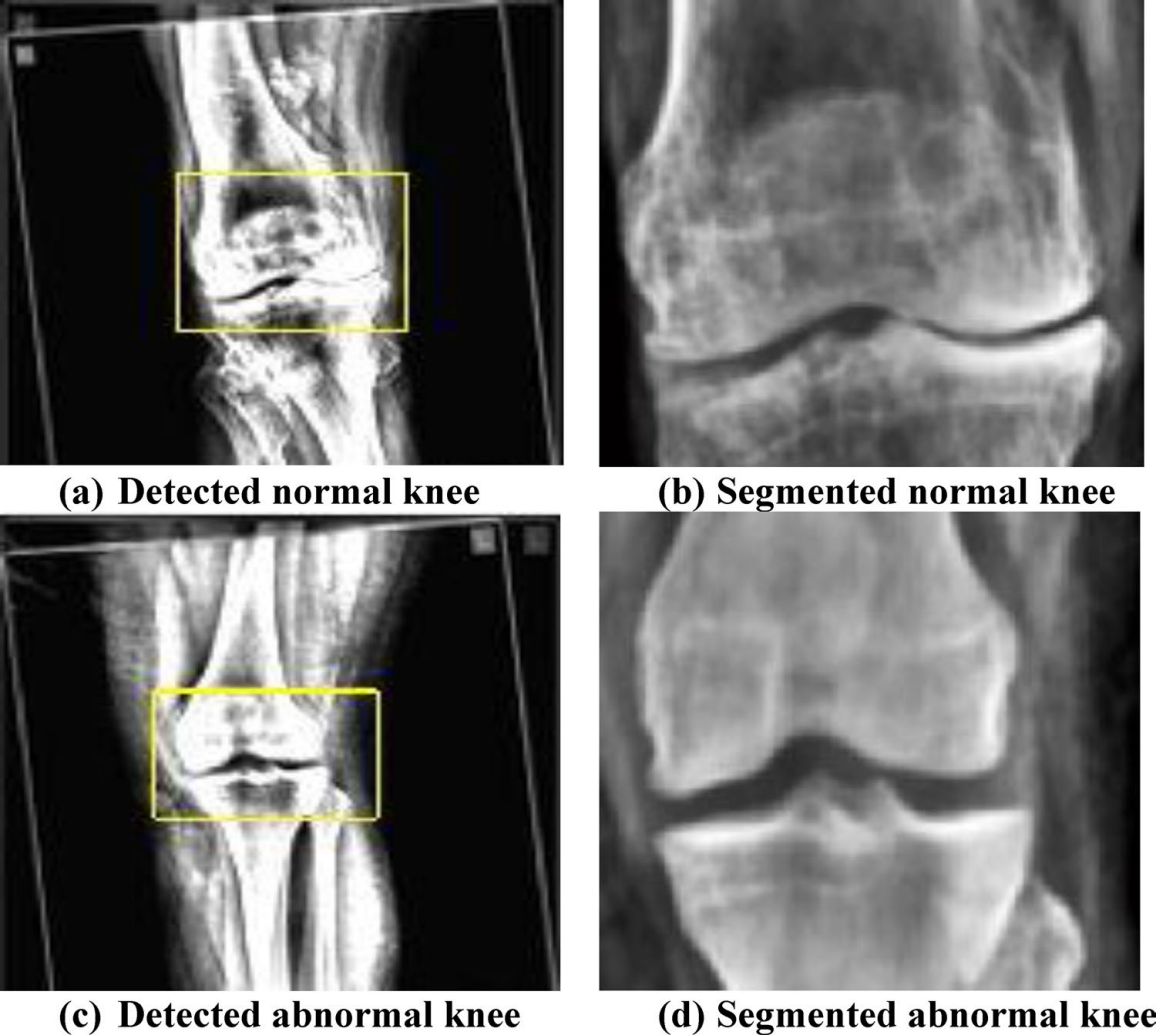
Detected and segmented images using the proposed method, (both normal and abnormal cases).

**Figure 9:**
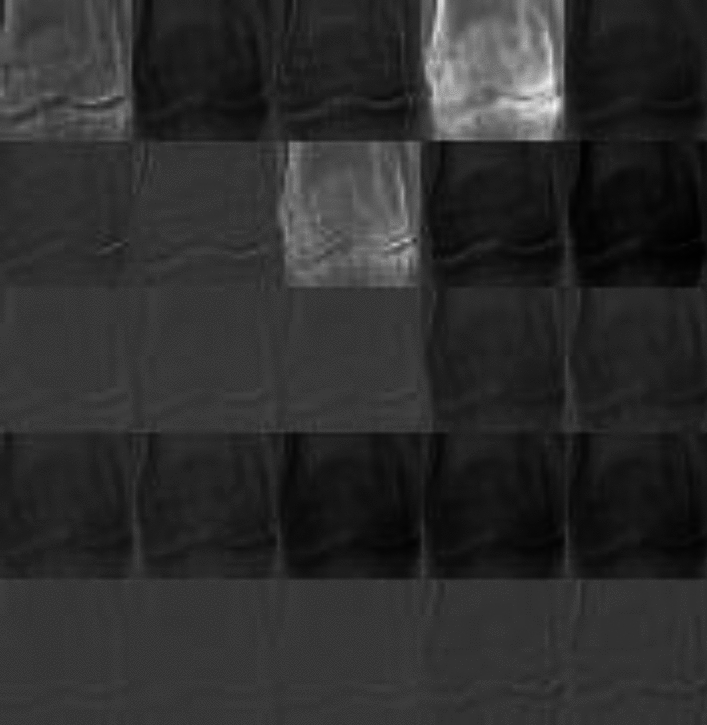
Integrated feature map image presented to FCNN of new DCNN model.

The proposed image fusion technique is applied and the fused images for both the normal and abnormal osteoarthritis images are as shown in Fig. 7. On performing the simulation process by presenting all the training and testing datasets, the detection and segmentation of the knee images are carried out. Figure 8 shows the simulated results of the detected image of the abnormal case and as well the segmented image of abnormal case and also the detected and segmented images of normal case. It is well observed from Fig. 8 that the modelled segmentation process activates for segmenting the knee joints perfectly in respect of both normal and abnormal cases. Segmentation accuracy (pixel accuracy) gives the overall accuracy of the segmentation algorithm and is the ratio of the number of correctly classified pixels to that of the total number of pixels pertaining to that image. For the presented knee images, segmentation accuracy was observed to be 90.65% for normal knee images and 93.51% for affected abnormal knee images. Table 6 presents the morphological features extracted and these features are used effectively for carrying out the classification using the new hybrid GAO-DCNN learning model. The integrated feature map image gets finally generated and is presented to the fully connected layer of the GAO based DCNN model. The final classification and grading of the segmented knee joints is carried out at this stage. Figure 9 provides the integrated feature map generated and is to be presented to the fully connected neural network (FCNN) layer of the propsoed DCNN model.

**Table 6:** Computed morphological feature values.

Morphological features	Corresponding Numerical values	
	Normal knee images	Abnormal knee images
Mean	1.89 ± 0.7	3.29 ± 0.38
Kurtosis	0.938*10^2^ ± 0.32	1.372*10^3^ ± 0.67
Skewness	0.382*10^4^ ± 0.17	4.291*10^4^ ± 0.85
Eccentricity	0.187 ± 0.37	2.198 ± 0.21
Major axis length	1.372 ± 0.2	4.392 ± 0.19
Minor axis length	0.382 ± 0.26	1.287 ± 0.87
Segmentation accuracy	90.65%	93.51%

Table 7 presents the knee detection rate evaluated using the proposed hybrid GAO-DCNN model. From the table, it is lucid that the modelled new optimized DCNN model correctly detects 252 normal knee images and obtains 99.6% of normal knee detection rate (NKDR). The proposed DCNN system correctly detects 202 Osteoarthritis knee images and obtains 99.5% of osteoarthritis knee detection rate (OKDR). Hence, the average knee detection rate is about 99.5% using the proposed DCNN architecture for the osteoarthritis detection system.

**Table 7:** Knee detection rate analysis using proposed approach.

Knee images count	Correctly detected knee images	Knee Detection Rate (KDR) in %
253 normal knee images	252 normal knee images	99.6% NKDR
203 Osteoarthritis knee images	202 Osteoarthritis knee images	99.5% OKDR
456 knee images	454 knee images	99.5% KDR

**Figure 10:**
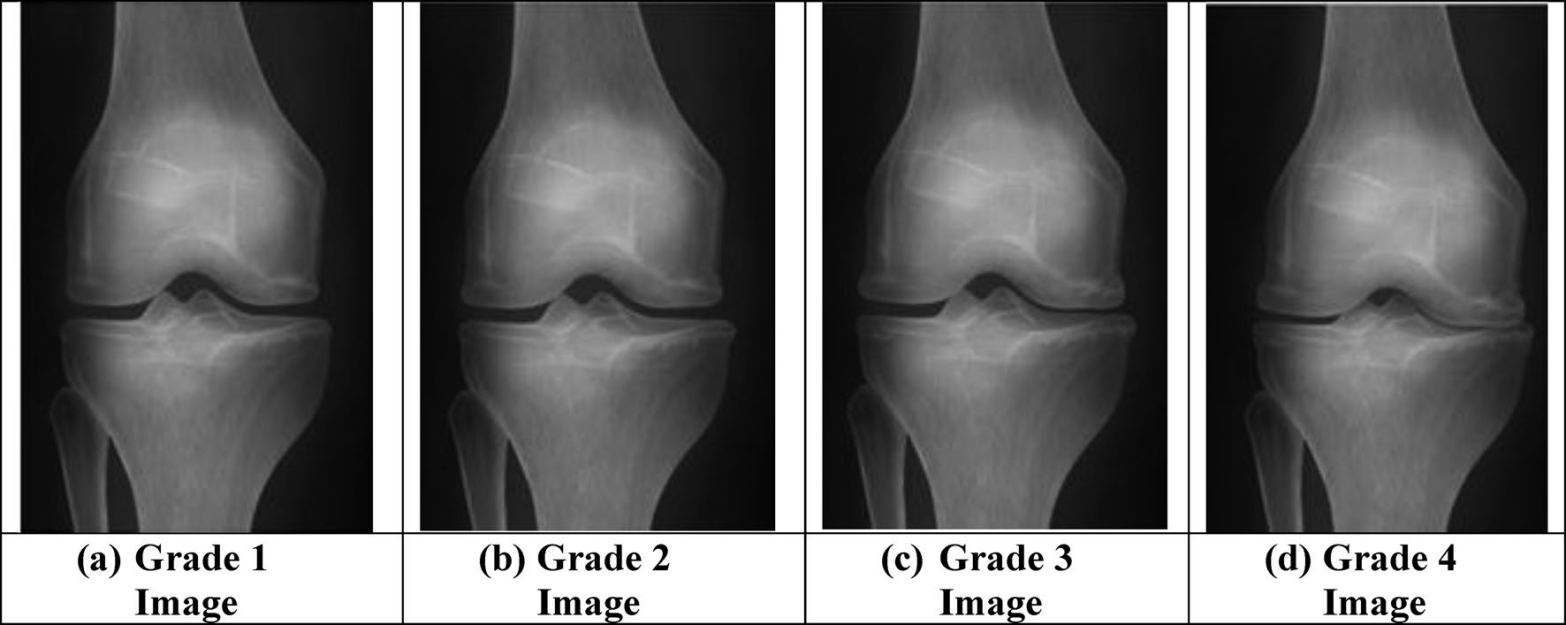
Classification outputs using proposed GAO-DCNN model.

Classification process is carried out using the proposed hybrid GAO-DCNN model and the classified abnormal osteoarthritis knee images are graded between Grade 1 to Grade 4 using the proposed dual CNN architecture in this study. The proposed optimized dual CNN architecture is trained by the morphological features extracted within the convolutional layers as the Grade 1 (Fig. 10a), Grade 2 (Fig. 10b), Grade 3 (Fig. 10c) and Grade 4 (Fig. 10d) segmented knee joint regions images to produce the trained patterns. In testing mode of the DCNN classifier, the morphological features of the segmented knee joint region of the test knee image are classified with respect to the trained patterns to identify the test abnormal knee image that is belonging to the specific Grade. The classified and graded knee images using the proposed method are illustrated in Fig. 10. The evaluation criterion of the performance metrics computed at the time of classification of knee images using the proposed GAO-DCNN is provided in Table 8. Table 9 provides the experimental results of the performance metrics evaluated during the classification of knee images using the proposed GAO-DCNN model.

**Table 8:** Evaluation criterion of the metrics computed.

Sl.No	Performance Metric	Evaluation Criterion
1	Sensitivity (Se)	$Se=\frac{TP}{TP+FN}$
2	Specificity (Sp)	$Sp=\frac{TN}{TN+FP}$
3	Classification Accuracy (Acc)	$Acc=\frac{TP+TN}{TP+TN+FP+FN}$

*TP* and *TN* are the correctly classified knee joint pixels and non-knee joint pixels respectively

*FP* and *FN* are the wrongly classified knee joint pixels and non-knee joint pixels respectively

**Table 9:** Performance metrics evaluated using new GAO-DCNN technique.

Normal Knee Images				Osteoarthritis Knee Images			
Normal cases	Se (%)	Sp (%)	Acc (%)	OA cases	Se (%)	Sp (%)	Acc (%)
N1	98.8	97.9	99.2	OA1	97.8	99.4	98.8
N2	98.9	98.9	98.7	OA2	98.3	99.3	98.4
N3	97.8	98.5	98.9	OA3	98.9	99.7	98.8
N4	97.5	98.8	98.4	OA 4	98.6	98.4	99.3
N5	98.9	98.5	98.7	OA5	97.9	98.9	99.2
N6	98.5	99.2	98.9	OA6	97.5	98.3	98.7
N7	98.7	99.4	98.5	OA7	98.8	99.6	98.4
N8	98.3	98.6	98.6	OA8	98.5	98.6	98.7
N9	98.2	98.3	98.3	OA9	97.9	98.4	98.5
N10	98.9	98.7	98.8	OA10	98.3	98.7	98.9
Average	98.45	98.68	98.7	Average	98.25	98.93	98.77

From Table 9, it is well elucidated that the proposed hybrid GAO-DCNN system for the detection of Osteoarthritis obtains 98.45% of Se, 98.68% of Sp and 98.7% of Acc for normal knee case- knee joint segmentation. The proposed GAO-DCNN system for the detection of Osteoarthritis obtains 98.25% of Se, 98.93% of Sp and 98.77% of Acc for abnormal knee case- knee joint segmentation. Table 10 presents the comparative analysis of knee joint detection systems in normal case with the conventional systems Liu et al.^3^, Tiulpin et al.^2^, Pingjun Chen et al. (2019), Ibraheem et al.^33^ and Abd El-Ghany et al.^35^. The detection time is the classification time consumed for detecting the normal knee image using the proposed method and it is measured in ms. From Table 10, the detection time of the proposed method is significantly less while compared with the other methods.

**Table 10:** Comparative analysis for Knee Images using proposed approach (Normal Cases).

Methods	Se (%)	Sp (%)	Acc (%)	ROC	Detection time (ms)	Mean Square Error (MSE)
Chen et al.^4^	95.19	95.09	96.28	0.74	1.9	3.1648
Liu et al.^3^	95.38	95.28	95.67	0.81	1.1	1.2249
Tiulpin et al.^2^	94.39	95.10	96.03	0.75	1.3	0.6132
Ibraheem et al.^33^	93.61	94.91	95.09	0.77	1.1	2.9187
Abd El-Ghany et al.^35^	96.22	96.37	96.91	0.83	1.0	1.0647
FFA-DCNN Classifier	88.67	92.16	91.35	0.70	1.5	3.2914
GWO-DCNN Classifier	90.63	93.15	92.06	0.72	1.2	2.6935
AQO-DCNN Classifier	96.48	95.36	96.59	0.87	0.9	0.5008
Proposed hybrid GAO-DCNN classifier	98.45	98.68	98.70	0.92	0.3	0.0057

Receiver Operating Characteristics (ROC) is the performance analysis metrics which is computed between sensitivity and 1-specificity. The value of ROC for the ideal case lies between 0.8 and 1. The ROC values of knee joint detection systems in normal case and abnormal case is depicted in the following tables to be nearer to 1, confirming the superiority of the proposed GAO-DCNN classifier. Tables 10 and 11 also presents the comparison made with respect to the optimization approaches. The proposed GAO-DCNN is compared with the Fire-Fly Algorithm (FFA)-DCNN, Grey Wolf Optimizer (GWO)-DCNN and AQO-DCNN and in all the cases the DCNN model developed remains the same and the parameter optimization is done with FFA, GWO and AQO. Gaussian mutation-based GAO has resulted in better results and confirm the effectiveness for the OA detection and grading.

**Table 11:** Comparative analysis for Knee Images using proposed approach (OA Cases).

Methods	Se (%)	Sp (%)	Acc (%)	ROC	Detection time (ms)	Mean Square Error (MSE)
Chen et al.^4^	94.47	95.98	95.26	0.78	2.1	3.2215
Liu et al.^3^	95.38	95.95	96.29	0.79	1.1	1.2768
Tiulpin et al.^2^	94.86	94.36	95.75	0.81	1.7	0.7039
Ibraheem et al.^33^	94.95	94.61	95.61	0.70	1.9	2.1378
Abd El-Ghany et al.^35^	96.00	96.93	96.22	0.83	1.0	1.1952
FFA-DCNN Classifier	93.29	92.44	94.00	0.75	1.5	4.0026
GWO-DCNN Classifier	92.78	93.05	93.69	0.72	1.3	3.9140
AQO-DCNN Classifier	95.49	94.19	96.48	0.86	0.9	0.5561
Proposed hybrid GAO-DCNN classifier	98.25	98.93	98.77	0.91	0.4	0.0091

Table 11 shows the comparative analysis of the knee joint detection systems in abnormal Osteoarthritis cases with the conventional systems Liu et al.^3^, Tiulpin et al.^2^, Pingjun Chen et al. (2019), Ibraheem et al.^33^ and Abd El-Ghany et al.^35^. It is clear from the comparative table, that the modelled new hybrid GAO based DCNN has resulted in better values of performance metrics than the existing techniques from the previous literatures for the same datasets. In Table 11, the detection time of the proposed method is significantly less when compared to all the other methods for abnormal case. From Tables 10 and 11, it can be well noted that the mean square error metric has been significantly reduced using the new GAO-DCNN model than all other compared techniques. This is due to the optimized weights used in the FCNN layer of the dual CNN using the Gaussian mutation based Aquila optimization algorithm.

Table 12 presents the analysis of the grading rates of the developed new GAO-DCNN learning model. The total number of OA knee images used in this research study is 813. These 813 abnormal OA images are split into 122 Grade 1 images, 138 Grade 2 images, 297 Grade 3 images and 256 Grade 4 images. The proposed system stated in this paper correctly graded 120 images in Grade 1 and obtains 98.3% GR, correctly graded 133 images in Grade 2 and obtains 96.3% GR, correctly graded 292 images in Grade 3 and obtains 98.3% GR and correctly graded 245 images in Grade 4 and obtains 95.7% GR. Therefore, the average grading rate (GR) of the proposed knee OA grading system is about 97.1% proving its efficacy in classification of osteoarthritis knee images. Table 13 shows the comparisons of grading rate of proposed method with other state of the art methods from previous literatures^17^. From this comparative analysis, the proposed OA grading system using GAO-DCNN provides higher GR due to its better architecture design and optimal weight tuning process of FCNN layer.

**Table 12:** Grading rate evaluation using proposed GAO-DCNN classifier.

OA Grades	Number of knee images	Correctly graded images	Incorrectly graded images	Grading Rate (GR) in %
Grade 1	122	120	2	98.3
Grade 2	138	133	5	96.3
Grade 3	297	292	5	98.3
Grade 4	256	245	11	95.7
Total	813	790	23	97.1

**Table 13:** Comparisons of grading rates of proposed classifier.

OA Grades	Grading Rate (GR) in %	
	Kellgren–Lawrence (KL) grading Wang et al.^17^	Proposed hybrid GAO-DCNN classifier
Grade 1	95.1	98.3
Grade 2	95.3	96.3
Grade 3	95.7	98.3
Grade 4	94.2	95.7

**Figure 11:**
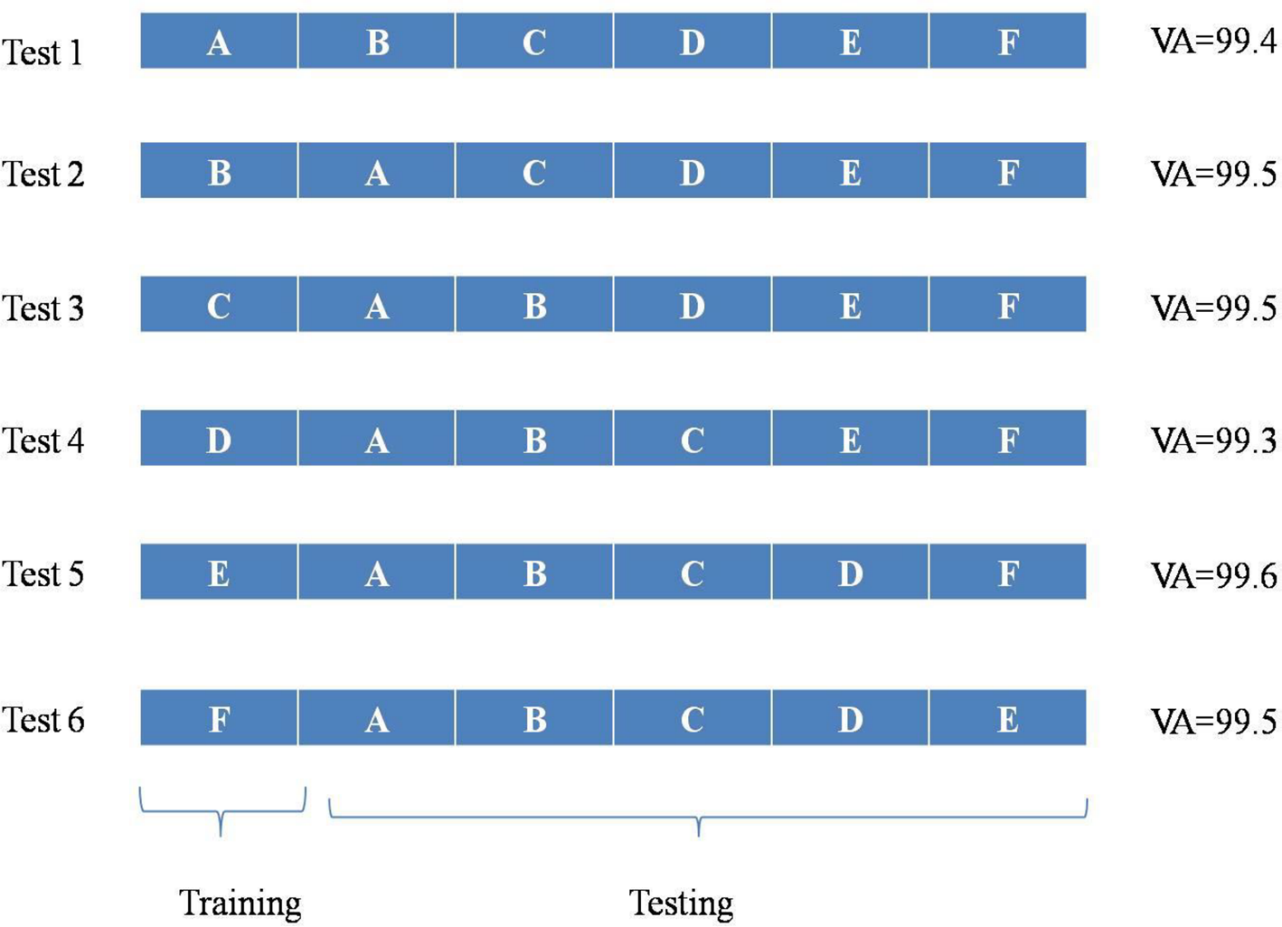
k-fold cross validation method for the proposed classifier.

The k-fold validation method (Mahesh et al.^24^) is employed in this paper to validate the performance of the proposed knee classification and grading system. Figure 11 shows the k-fold validation method for the proposed knee grading system. In this work, 5 folds are used and hence the value of K is 5, which splits the entire 1827 dataset images into six modules A to F, as depicted in Fig. 11. Each module in each fold contains 366 knee images. In test 1, module A is tested, where the remaining modules are trained and the Validation Accuracy (VA) 99.4% is obtained. In test 2, module B is tested, where the remaining modules are trained and the VA 99.5% is obtained. In test 3, module C is tested, where the remaining modules are trained and the VA 99.5% is obtained. In test 4, module D is tested, where the remaining modules are trained and the VA 99.3% is obtained.

In test 5, module E is tested, where the remaining modules are trained and the VA 99.6% is obtained. In test 6, module F is tested, where the remaining modules are trained and the VA 99.5% is obtained. The training accuracy is about 99.4% for the proposed knee grading system. In this work, 25 epochs are used for both training and validation in k-fold algorithm. The average VA 99.5% is obtained using k-fold validation method, which is similar to the KDR of the proposed system. Therefore, it is concluded that the proposed GAO-DCNN knee grading system in this work provides higher KDR. The proposed new DCNN algorithm, which is a deep learning algorithm using two stack of convolutional neural network is executed to detect and classify the osteoarthritis affected patients. In the novel DCNN model is optimized for its weight and bias coefficients using the novel Gaussian aquila optimizer, this optimized parameter coefficients enables the DCNN model to converge with better accuracy rate and minimized mean square error value. The layer design of the DCNN ensures highly significant filtering, feature extraction and pooling along with necessary drop outs, thereby resulting in better grading rate.

Table 14 presents the complexity analysis done for the existing methods and that of the proposed GAO-DCNN classifier for the same knee image datasets. The computational complexity has been significantly reduced using the proposed hybrid GAO-DCNN classifier compared to that of the other methods^16,26,33,35^ with respect to the depth of parameters and other parameters considered. This is due to the designed layer structure of the DCNN and the optimization GAO algorithm used in computing the optimal weight values between the deep layers. Henceforth, the batch size, generations and computational time are prominently decreased proving the superiority of the proposed GAO-DCNN classifier model.

**Table 14:** Complexity analysis of the proposed model with existing techniques.

Techniques	Depth	# No. of Parameters	Computational Time (ms)	Batch size	Epochs
Alex Net^16^	138	1.39 M	9.02	48	576
Google Net^16^	1048	11.6 M	12.68	36	894
ResNet34^16^	151	1.5 M	8.11	132	708
ResNet152^16^	67	9.3 M	57.63	132	246
Pre-ResNet^26^	98	31.6 M	41.29	72	540
Wide-ResNet^26^	123	6.3 M	19.67	64	196
DenseNet^26^	56	18.6 M	7.33	48	132
Ibraheem et al.^33^	64	4.7 M	1.90	48	110
Abd El-Ghany et al.^35^	36	1.2 M	1.00	48	98
Dual CNN model	42	4.2 M	3.41	32	68
FFA-DCNN Classifier	31	0.96 M	1.5	28	106
GWO-DCNN Classifier	31	0.89 M	1.3	28	87
AQO-DCNN Classifier	31	0.79 M	0.9	24	61
Proposed GAO-DCNN classifier model	31	0.79 M	0.4	24	42

The developed GAO-DCNN model has addressed the limitations of the existing models in such a way that based on the input X-ray images provided, the features based on the joint space of the lateral and medial compartments gets included. DCNN model operates in such a way to include all the prominent features and also few approaches had inconsistency with respect to original description of images. Here the optimized version of GAO-DCNN is capable of maintaining the balance in the learning process with the architecture and layer design and thus avoids inconsistency if any. Earlier models were having separate filtering and feature extraction mechanisms and devising DCNN model performs the noise removal and feature extraction by itself thereby avoiding the difficulty in locating the definite osteophytes and overcomes discrimination of joint space narrowing. The model is capable of recognizing distinct arthritis also, such that it has evolved the better classification accuracy.

## Conclusion

A novel Gaussian mutation and momentum control factor based Gaussian Aquila Optimizer has been developed in this research study for determining the optimal solution for any parameters of concern. Along with the developed GAO approach, a new dual convolutional neural network has been modelled for performing the learning and classification process. The modelled new DCNN algorithm, which is a deep learning algorithm using two stack of convolutional neural network is executed to detect and classify the osteoarthritis affected patients. New DCNN model is optimized for its weight and bias coefficients using the novel Gaussian Aquila optimizer, this optimized parameter coefficients enables the DCNN model to converge with better accuracy rate and minimized mean square error value. Human knee X-ray images were presented to the proposed GAO-DCNN and the experimentation during the training and testing phase has resulted in better classification accuracy and grading rate compared to all other algorithms from previous literatures for the same data samples. The design of dual convolutional neural learning model has enriched in even detecting the edge features most accurately, thereby no loss of information of the X-ray images happens. Layered structure of DCNN has evolved to perform the training phase and testing phase individually, thereby the trained matrix of training phase was the initial weight for the testing phase and thereby the learning has progressed successfully. Grading of osteoarthritis has also been done and this has resulted in attaining grading rate of 97.1% proving its superiority over other compared models from previous literatures for same datasets. Computational complexity also has been significantly reduced using the modelled new hybrid GAO-DCNN classifier model.

The limitation of this research study is that, the proposed model has to be trained and tested with even a lot number of knee image samples to get higher accuracy rate. Also, the design of individual CNN models is to be done to decrease the depth of layers and number of parameters used during learning. Both these observed limitations shall be handled in future research study with increased number of knee X-ray datasets and also with knee MRI datasets.

## Data Availability

The datasets used and/or analysed during the current study shall be available from the corresponding author on reasonable request.

## References

[1] Thomas, K. A. *et al.* Automated classification of radiographic knee osteoarthritis severity using deep neural networks. *Radiol. Artif. Intell.* **2**(2), e190065 (2020). 32280948 10.1148/ryai.2020190065 PMC7104788

[2] Tiulpin, A. & Saarakkala, S. Automatic grading of individual knee osteoarthritis features in plain radiographs using deep convolutional neural networks. *Diagnostics* **10**(11), 932 (2020). 33182830 10.3390/diagnostics10110932 PMC7697270

[3] Liu, B., Luo, J. & Huang, H. Toward automatic quantification of knee osteoarthritis severity using improved Faster R-CNN. *Int. J. Comput. Assis. Radiol. Surg.* **15**, 457–466 (2020). 10.1007/s11548-019-02096-9 31938993

[4] Chen, P., Gao, L., Shi, X., Allen, K. & Yang, L. Fully automatic knee osteoarthritis severity grading using deep neural networks with a novel ordinal loss. *Comput. Med. Imag. Graph.* **75**, 84–92 (2019). 10.1016/j.compmedimag.2019.06.002 PMC9531250 31238184

[5] Kotti, M., Duffell, L. D., Faisal, A. A. & McGregor, A. H. Detecting knee osteoarthritis and its discriminating parameters using random forests. *Med. Eng. Phys.* **43**, 19–29 (2017). 28242181 10.1016/j.medengphy.2017.02.004 PMC5390773

[6] Chen, P. Knee osteoarthritis severity grading dataset. Mendeley Data, v1 (2018).

[7] Feichtinger, H.G. Advances in Gabor analysis. Springer Science & Business Media. Handbook of Mathematical Methods in Imaging. Springer, New York (2003).

[8] Palanisamy, G., Ponnusamy, P. & Gopi, V. P. An improved luminosity and contrast enhancement framework for feature preservation in color fundus images. *Signal Image Video Process.* **13**, 719–726 (2019). 10.1007/s11760-018-1401-y

[9] Lian, M. J. & Huang, C. L. Texture feature extraction of gray-level co-occurrence matrix for metastatic cancer cells using scanned laser pico-projection images. *Lasers Med. Sci.* **34**, 1503–1508 (2019). 30043142 10.1007/s10103-018-2595-5

[10] Alexos, A., Kokkotis, C., Moustakidis, S., Papageorgiou, E., & Tsaopoulos, D. Prediction of pain in knee osteoarthritis patients using machine learning: Data from Osteoarthritis Initiative. In *2020 11th International Conference on Information, Intelligence, Systems and Applications (IISA*) (pp. 1–7). IEEE (2020).

[11] Jamshidi, A. *et al.* Machine learning–based individualized survival prediction model for total knee replacement in osteoarthritis: data from the osteoarthritis initiative. *Arthritis Care Res.* **73**(10), 1518–1527 (2021). 10.1002/acr.24601 33749148

[12] Teoh, Y.X., Lai, K.W., Usman, J., Goh, S.L., Mohafez, H., Hasikin, K., Qian, P., Jiang, Y., Zhang, Y., & Dhanalakshmi, S. Discovering knee osteoarthritis imaging features for diagnosis and prognosis: Review of manual imaging grading and machine learning approaches. *J. Healthcare Eng.* (2022). 10.1155/2022/4138666 PMC8881170 35222885

[13] Hafezi-Nejad, N. *et al.* Prediction of medial tibiofemoral compartment joint space loss progression using volumetric cartilage measurements: data from the FNIH OA biomarkers consortium. *Eur. Radiol.* **27**, 464–473 (2017). 27221563 10.1007/s00330-016-4393-4

[14] Tolpadi, A. A., Lee, J. J., Pedoia, V. & Majumdar, S. Deep learning predicts total knee replacement from magnetic resonance images. *Sci. Rep.* **10**(1), 6371 (2020). 32286452 10.1038/s41598-020-63395-9 PMC7156761

[15] Guan, B. *et al.* Deep learning risk assessment models for predicting progression of radiographic medial joint space loss over a 48-MONTH follow-up period. *Osteoarthritis Cartilage* **28**(4), 428–437 (2020). 32035934 10.1016/j.joca.2020.01.010 PMC7137777

[16] Yeoh, P. S. Q. *et al.* Emergence of deep learning in knee osteoarthritis diagnosis. *Comput. Intell. Neurosci.* **2021**, 1–20 (2021). 10.1155/2021/4931437 PMC8598325 34804143

[17] Wang, Y., Wang, X., Gao, T., Du, L. & Liu, W. An automatic knee osteoarthritis diagnosis method based on deep learning: data from the osteoarthritis initiative. *J. Healthcare Eng.* **2021**, 1–10 (2021). 10.1155/2021/4310648 PMC8490030 34616534

[18] Kokkotis, C., Ntakolia, C., Moustakidis, S., Giakas, G. & Tsaopoulos, D. Explainable machine learning for knee osteoarthritis diagnosis based on a novel fuzzy feature selection methodology. *Phys. Eng. Sci. Med.* **45**(1), 219–229 (2022). 35099771 10.1007/s13246-022-01106-6 PMC8802106

[19] Debi, R. *et al.* Differences in gait patterns, pain, function and quality of life between males and females with knee osteoarthritis: a clinical trial. *BMC Musculoskeletal Disorders* **10**, 1–10 (2009). 19825163 10.1186/1471-2474-10-127 PMC2765955

[20] Favre, J., Erhart-Hledik, J. C. & Andriacchi, T. P. Age-related differences in sagittal-plane knee function at heel-strike of walking are increased in osteoarthritic patients. *Osteoarthritis Cartilage* **22**(3), 464–471 (2014). 24445065 10.1016/j.joca.2013.12.014 PMC4211113

[21] Kobsar, D., Osis, S. T., Boyd, J. E., Hettinga, B. A. & Ferber, R. Wearable sensors to predict improvement following an exercise intervention in patients with knee osteoarthritis. *J. Neuroeng. Rehabil.* **14**(1), 1–10 (2017). 28899433 10.1186/s12984-017-0309-z PMC5596963

[22] Rutherford, D. J. & Baker, M. Knee moment outcomes using inverse dynamics and the cross product function in moderate knee osteoarthritis gait: a comparison study. *J. Biomech.* **78**, 150–154 (2018). 30049451 10.1016/j.jbiomech.2018.07.021

[23] Pirker, W. & Katzenschlager, R. Gait disorders in adults and the elderly: a clinical guide. *Wiener Klinische Wochenschrift* **129**(3–4), 81–95 (2017). 27770207 10.1007/s00508-016-1096-4 PMC5318488

[24] Mahesh, T.R., Dhilip Kumar, V., Vinoth Kumar, V., Asghar, J., Geman, O., Arulkumaran, G., & Arun, N. AdaBoost ensemble methods using K-fold cross validation for survivability with the early detection of heart disease. *Comput. Intell. Neurosci*. (2022). 10.1155/2022/9005278 PMC9038394 35479597

[25] Chan, L. C., Li, H. H. T., Chan, P. K. & Wen, C. A machine learning-based approach to decipher multi-etiology of knee osteoarthritis onset and deterioration. *Osteoarthritis and Cartilage Open* **3**(1), 100135 (2021). 36475069 10.1016/j.ocarto.2020.100135 PMC9718099

[26] Gan, H. S., Ramlee, M. H., Wahab, A. A., Lee, Y. S. & Shimizu, A. From classical to deep learning: review on cartilage and bone segmentation techniques in knee osteoarthritis research. *Artif. Intell. Rev.* **54**(4), 2445–2494 (2021). 10.1007/s10462-020-09924-4

[27] Shoaib, M. A. *et al.* Speckle noise diffusion in knee articular cartilage ultrasound images. *Current medical imaging* **16**(6), 739–751 (2020). 32723246 10.2174/1573405615666190903143330

[28] Collins, J. E., Neogi, T. & Losina, E. Trajectories of structural disease progression in knee osteoarthritis. *Arthritis Care Res.* **73**(9), 1354–1362 (2021). 10.1002/acr.24340 PMC7710564 32491247

[29] Siaton, B. C., Hogans, B. H. & Hochberg, M. C. Precision medicine in osteoarthritis: not yet ready for prime time. *Expert Rev. Precis. Med. Drug Develop.* **6**(1), 5–8 (2021). 10.1080/23808993.2020.1842731

[30] Jamshidi, A., Leclercq, M., Labbe, A., Pelletier, J.P., Abram, F., Droit, A., & Martel-Pelletier, J. Identification of the most important features of knee osteoarthritis structural progressors using machine learning methods. *Therapeut. Adv. Musculoskeletal Dis*. 12, 1759720X20933468 (2020). 10.1177/1759720X20933468 PMC7427139 32849918

[31] Teoh, Y. X., Othmani, A., Lai, K. W., Goh, S. L. & Usman, J. Stratifying knee osteoarthritis features through multitask deep hybrid learning: data from the osteoarthritis initiative. *Comput. Methods Programs Biomed.* **242**, 107807 (2023). 37778138 10.1016/j.cmpb.2023.107807

[32] Kijowski, R., Fritz, J., & Deniz, C.M. Deep learning applications in osteoarthritis imaging. *Skeletal Radiol*. 1–14 (2023). 10.1007/s00256-023-04296-6 PMC10409879 36759367

[33] Ibraheem, M. R., Almuayqil, S. N., Abd El-Aziz, A. A., Tawfeek, M. A. & Talaat, F. M. Diagnosis of patellofemoral osteoarthritis using enhanced sequential deep learning techniques. *Egypt. Inf. J.* **24**(3), 100391 (2023).

[34] Hu, J. *et al.* DeepKOA: a deep-learning model for predicting progression in knee osteoarthritis using multimodal magnetic resonance images from the osteoarthritis initiative. *Quantitat. Imag. Med. Surg.* **13**(8), 4852 (2023). 10.21037/qims-22-1251 PMC10423358 37581080

[35] Abd El-Ghany, S., Elmogy, M. & Abd El-Aziz, A. A. A fully automatic fine-tuned deep learning model for knee osteoarthritis detection and progression analysis. *Egypt. Inf. J.* **24**(2), 229–240 (2023).

[36] Mahum, R., Irtaza, A., El-Meligy, M.A., Sharaf, M., Tlili, I., Butt, S., Mahmood, A., & Awais, M. A robust framework for severity detection of knee osteoarthritis using an efficient deep learning model. *Int. J. Pattern Recognit. Artif. Intell*, 2352010 (2023).

[37] Abualigah, L. *et al.* Aquila optimizer: a novel meta-heuristic optimization algorithm. *Comput. Indus. Eng.* **157**, 107250 (2021). 10.1016/j.cie.2021.107250

[38] Zhao, J., Gao, Z. M. & Chen, H. F. The simplified Aquila optimization algorithm. *IEEE Access* **10**, 22487–22515 (2022). 10.1109/ACCESS.2022.3153727

[39] Sasmal, B., Hussien, A.G., Das, A., & Dhal, K.G. A comprehensive survey on Aquila optimizer. *Arch. Comput. Methods Eng.* 1–28 (2023). 10.1007/s11831-023-09945-6 PMC10245365 37359742

[40] Huang, C., Huang, J., Jia, Y. & Xu, J. A hybrid Aquila optimizer and its K-means clustering optimization. *Trans. Inst. Measur. Control* **45**(3), 557–572 (2023). 10.1177/01423312221111607

